# *Cul3* and the BTB Adaptor *Insomniac* Are Key Regulators of Sleep Homeostasis and a Dopamine Arousal Pathway in Drosophila

**DOI:** 10.1371/journal.pgen.1003003

**Published:** 2012-10-04

**Authors:** Cory Pfeiffenberger, Ravi Allada

**Affiliations:** Department of Neurobiology, Northwestern University, Evanston, Illinois, United States of America; University of Pennsylvania, United States of America

## Abstract

Sleep is homeostatically regulated, such that sleep drive reflects the duration of prior wakefulness. However, despite the discovery of genes important for sleep, a coherent molecular model for sleep homeostasis has yet to emerge. To better understand the function and regulation of sleep, we employed a reverse-genetics approach in *Drosophila*. An insertion in the BTB domain protein *CG32810/insomniac* (*inc*) exhibited one of the strongest baseline sleep phenotypes thus far observed, a ∼10 h sleep reduction. Importantly, this is coupled to a reduced homeostatic response to sleep deprivation, consistent with a disrupted sleep homeostat. Knockdown of the INC-interacting protein, the E3 ubiquitin ligase *Cul3*, results in reduced sleep duration, consolidation, and homeostasis, suggesting an important role for protein turnover in mediating INC effects. Interestingly, *inc* and *Cul3* expression in post-mitotic neurons during development contributes to their adult sleep functions. Similar to flies with increased dopaminergic signaling, loss of *inc* and *Cul3* result in hyper-arousability to a mechanical stimulus in adult flies. Furthermore, the *inc* sleep duration phenotype can be rescued by pharmacological inhibition of tyrosine hydroxylase, the rate-limiting enzyme for dopamine biosynthesis. Taken together, these results establish *inc* and *Cul3* as important new players in setting the sleep homeostat and a dopaminergic arousal pathway in *Drosophila*.

## Author Summary

Sleep is an essential behavior that encompasses roughly a third of our lives; however, the underlying function remains a mystery. The fruit fly has emerged as an important model system for understanding sleep behavior, exhibiting several behavioral and genetic similarities with mammalian sleep, including consolidated immobility, an elevation of arousal threshold to a range of stimuli, homeostatic drive, and manipulation by proven stimulants and sedatives. We tested disruptions of candidate sleep genes and identified a gene called *insomniac* that exhibits one of the strongest and most robust sleep phenotypes to date, including a suppressed homeostatic response to sleep deprivation. We find similar phenotypes for a gene previously shown to interact with *inc* and a known regulator of protein degradation, *Cul3*, linking sleep homeostasis to protein turnover. Importantly, we find that *insomniac* functions in a known arousal system in the brain, as defined by the neurotransmitter dopamine. This work provides an important insight into the genetic basis of sleep homeostasis with the discovery of a new molecular component of a dopaminergic arousal pathway. Given the conservation of fly and mammalian systems, these studies may lead to new insights into the molecules that mediate sleep homeostasis and arousal in humans.

## Introduction

Sleep is a homeostatically regulated process, consuming roughly one-third of our lives, yet its function remains a mystery. To identify novel pathways governing sleep, we and others have employed a genetic approach in *Drosophila*. The fruit fly shares several core features of sleep with its mammalian counterparts, including behavioral quiescence, reduced responsiveness to sensory stimuli, and homeostatic responses to sleep deprivation [1], [2]. To date, several forward-genetics screens have been performed, successfully identifying mutants that increase or decrease sleep duration to varying degrees, highlighting the roles of **(1)** membrane excitability via the *Shaker* potassium channel [3]–[6], **(2)** neurotransmitters such as dopamine [7]–[10], **(3)** growth factors such as *epidermal growth factor* [11], and **(4)** signal transduction pathways among others [12]–[14]. Of these mutations, those affecting *Shaker* or dopamine yield the most robust phenotypes [4]–[6], [9], [15]. Yet how these key pathways regulate sleep homeostasis remains unclear.

Here we report the result of a reverse-genetics approach aimed at identifying regulators of sleep and arousal in *Drosophila*. We focused on the gene with the most robust phenotype, *insomniac* (*inc*), a target identifier for the E3 ubiquitin ligase *Cullin-3* (*Cul3*) [16]. We find that flies lacking *inc* or *Cul3* exhibit strikingly reduced and poorly consolidated sleep. Developmental expression of *inc* and *Cul3* in post-mitotic neurons contributes to these adult sleep phenotypes. In addition to their baseline sleep phenotypes, both *inc* and *Cul3* also exhibit reduced homeostatic responses to sleep deprivation as well as hyper-arousability to mechanical stimuli. Baseline sleep in flies deficient for *inc* or *Cul3* can be rescued by pharmacological inhibition of dopamine synthesis, but are behaviorally resistant to pharmacologically increased dopamine synthesis, consistent with the hypothesis that these genes operate in a dopamine arousal pathway. Taken together, our data indicate a central role for *inc* and *Cul3* in sleep homeostasis and dopamine-mediated arousal.

## Results

### A reverse-genetics screen for sleep genes

**Figure 1:**
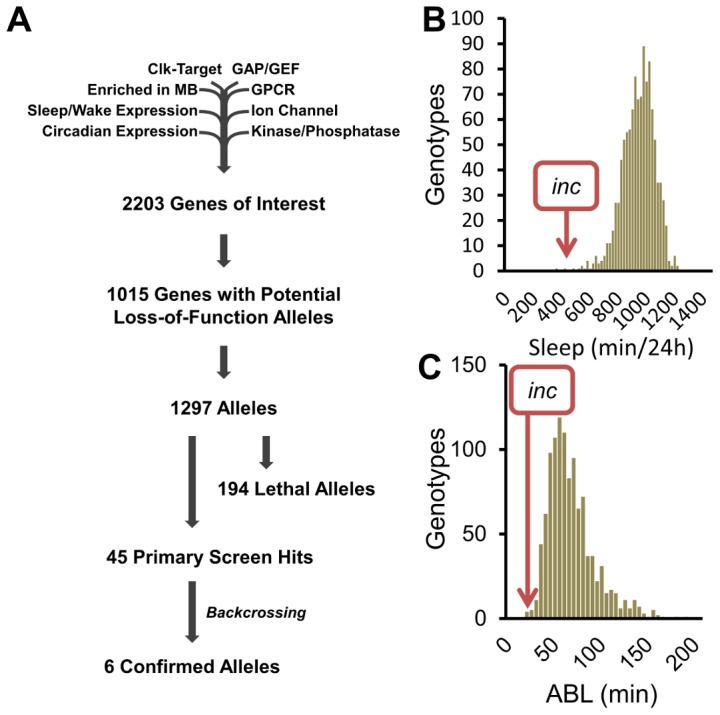
A reverse-genetics screen identifies novel sleep genes. (A) Flow chart shows gene selection at each stage of the screen. (B) The sleep duration and (C) average sleep bout length (ABL) distributions from the reverse-genetics screen.

To identify novel sleep genes, we performed a reverse-genetics screen, focusing on genes previously reported to have sleep/wake-dependent expression [3], [17], circadian expression [3], [18], *Clk*-target genes [19], kinases/phosphatases, GTPase-activating proteins, guanine nucleotide exchange factors, G-protein coupled receptors, ion channels, and synaptic components (Flybase). Of the initial 2203 genes, we were able to analyze 1015 with potential loss-of-function alleles (Figure 1A). To compensate for potential differences in genetic background, previously existing alleles were tested over a deficiency (Df) from the isogenic DrosDel collection [20], and allele/Df combinations shifted at least 2 standard deviations from the population mean for sleep duration and/or average sleep bout length (ABL) in males in 3 separate behavior experiments were considered hits. In the case of X-linked genes, allele virgins were crossed to X-linked deficiency males and the F1 allele/Y males were tested.

At the conclusion of the primary screen we identified 45 alleles with reproducible sleep duration or bout length phenotypes (Figure 1A–1C). To determine the influence of genetic background on these phenotypes we backcrossed the 45 allele hits for 5 generations into the isogenic *iso31* background developed by DrosDel [20]. Surprisingly, despite outcrossing the alleles to isogenic Df lines in the primary screen, only 6 of the hits retained their sleep phenotypes after backcrossing (Figure 1A). For example, in the primary screen we identified the following insertion alleles as having a striking effect on sleep behavior: (1) *mXr^DG17503^* exhibited increased sleep duration, (2) *CG9135^f03307^* had increased ABL, and (3) *RhoGDI^EY02738^* resulted in reduced sleep (Figure S1A–S1C). However, after backcrossing into the *iso31* background the sleep phenotypes are no longer observable (Figure S1A–S1C). To distinguish between a potential suppressor in the *iso31* background and a flanking sleep mutant in the original *RhoGDI^EY02738^* background, we analyzed sleep in precise excisions of the *EY02738* transposon. Importantly, we found that the *RhoGDI^EY02738^* short-sleep phenotype persists after precise excision of the P-element, suggesting that a distinct mutation in this background is responsible for the phenotype. Taken together, these observations highlight the important modulatory effect genetic background has on sleep. Furthermore, these results make clear that simply outcrossing an allele to a deficiency line is insufficient to rule out genetic background as a primary cause of phenotype. Importantly, these results do not exclude a role for sleep regulation for the 39 primary screen hits that do not retain a sleep phenotype after backcrossing, as either the *iso31* or the original background may have a modifier that enhances or suppresses the sleep phenotype. Future work will be required to confirm a sleep regulatory role for these alleles.

### The homeostatic regulation of sleep is disrupted in *insomniac* mutants

**Figure 2:**
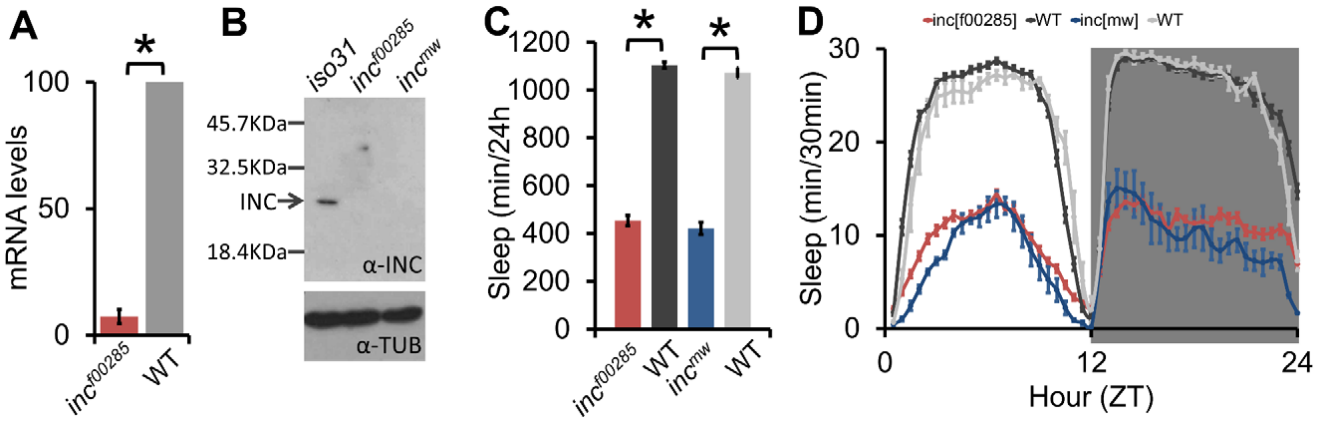
Sleep is reduced throughout the day in *inc* mutants. (A) *inc* mRNA transcript levels are reduced in *inc^f00285^* (n = 4×20 male heads/genotype). (B) Western blot shows INC protein is detectable in wild type (*iso31*), but not *inc^f00285^* or *inc^mw^* male heads (n = 30 male heads/genotype). (C) Sleep duration in *inc^f00285^* (red, n = 44 males), *inc^f00285^* isogenic control (WT, dark grey, n = 47 males), *inc^mw^* (blue, n = 72 males), and *inc^mw^* isogenic control (WT, light grey, n = 32 males). (D) Sleep duration per 30 min bins. Data is shown as 30 min periods averaged from 4 days 12 h∶12 h light∶dark (ZT0 is lights-on, ZT12 is lights-off) in *inc^f00285^* (red, n = 88 males), *inc^f00285^* isogenic control (WT, dark grey, n = 47 males), *inc^mw^* (blue, n = 72 males), and *inc^mw^* isogenic control (WT, light grey, n = 32 males), showing that *inc* mutant flies exhibit reduced sleep throughout a 24 h day and anticipatory locomotor activity before lights-on and –off. Error bars are SEM. * p<0.005 with Student's t test.

Despite the influence of genetic background, we were able to identify one allele with a robust and reproducible sleep reduction even after backcrossing: *f00285*, a *piggyBac* insertion in the 5′ untranslated region of *insomniac* (*inc*, *CG32810)* (Figure 1B–1C, Figure S2A), a gene selected for its BTB protein-protein interaction domain and recently linked to sleep regulation [21]. To complement this allele we created a second allele by knocking in a *miniwhite* gene just upstream of the *inc* stop codon (Figure S2A, *inc^mw^*). After backcrossing, expression of *inc* transcript in *inc^f00285^* flies was nearly undetectable (Figure 2A); furthermore, although we do not expect *inc^mw^* to affect transcript levels given the insertion location, we observed that INC protein was undetectable in both *inc^f00285^* and *inc^mw^* (Figure 2B) indicating that the insertions strongly disrupt *inc* function. We found that these backcrossed *inc* alleles sleep greater than 600 minutes less than their isogenic control flies (Figure 2C–2D).

To further show the sleep relevant phenotypes were due to a disruption of *inc*, we demonstrated that sleep duration and bout length phenotypes could be rescued with Y-linked genomic duplications that included the *inc* genomic region, but not with a Y-linked duplication from the same collection that did not contain *inc* (Figure S2A–S2D). Furthermore, we found that a deletion that removes *inc*, as well as *inc* transheterozygotes, failed to complement the recessive *inc* phenotype (Figure S3). *inc* mutants displayed reduced sleep during both light and dark periods with increases in locomotor activity; nonetheless, their sleep levels dropped in anticipation of light-dark and dark-light transitions, consistent with intact circadian clock function (Figure 2D). These effects on sleep duration are comparable to those observed for the *Sh^mns^* allele [15] in the *iso31* background under our conditions (data not shown) and represent one of the strongest sleep phenotypes thus far observed in *Drosophila* or any animal model.

**Figure 3:**
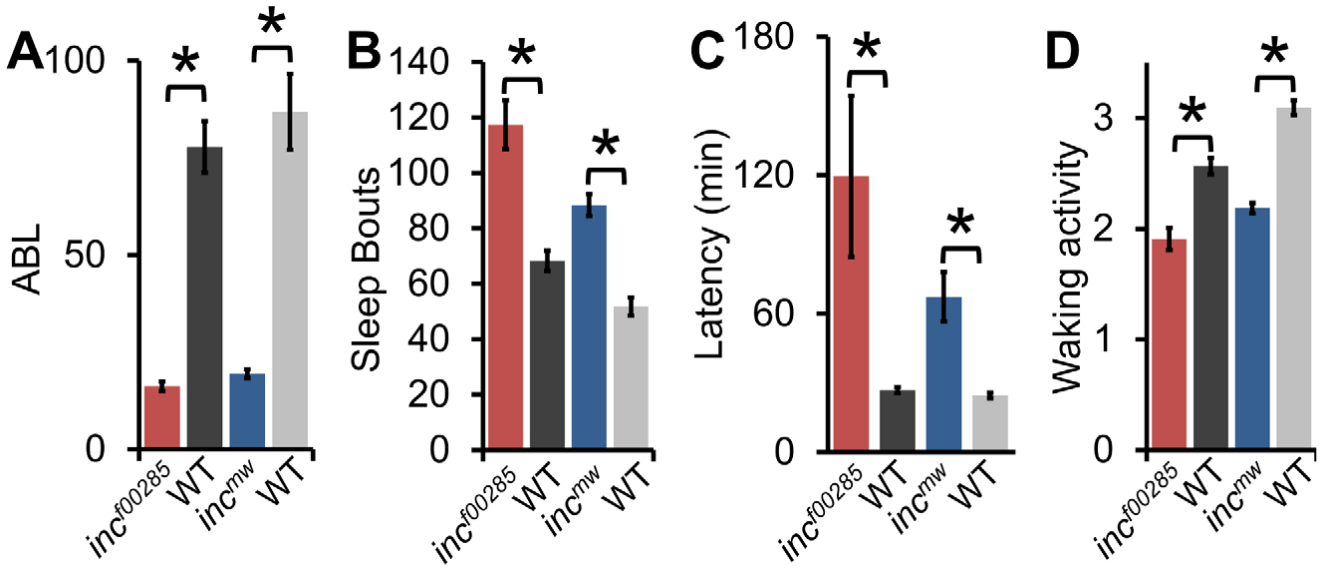
Sleep architecture is altered in *inc* mutants. (A) Average sleep bout length (ABL), (B) total number of sleep bouts, (C) latency to sleep after lights-off, (D) and waking activity in *inc^f00285^* (red, n = 44 males), *inc^f00285^* isogenic control (WT, dark grey, n = 47 males), *inc^mw^* (blue, n = 72 males), and *inc^mw^* isogenic control (WT, light grey, n = 32 males). Error bars are SEM. * p<0.005 with Student's t test for all data except sleep bout length. Sleep bout length is not normally distributed; therefore, Mann-Whitney U Test was used.

The sleep reduction is associated with specific changes in sleep architecture. First, *inc* flies displayed a decrease in sleep bout length (Figure 3A) that was accompanied by an increase in sleep bout number (Figure 3B), suggesting that flies were repeatedly attempting to initiate sleep but were unable to maintain it. Flies, like humans, typically fall asleep rapidly after the lights turn-off. However, *inc* files exhibited an increased latency to sleep after lights-off (Figure 3C). Despite the dramatic reduction in sleep levels, *inc* flies were not hyperactive, instead displaying a modest reduction in activity during wakefulness, suggesting a primary effect on sleep rather than activity (Figure 3D).

**Figure 4:**
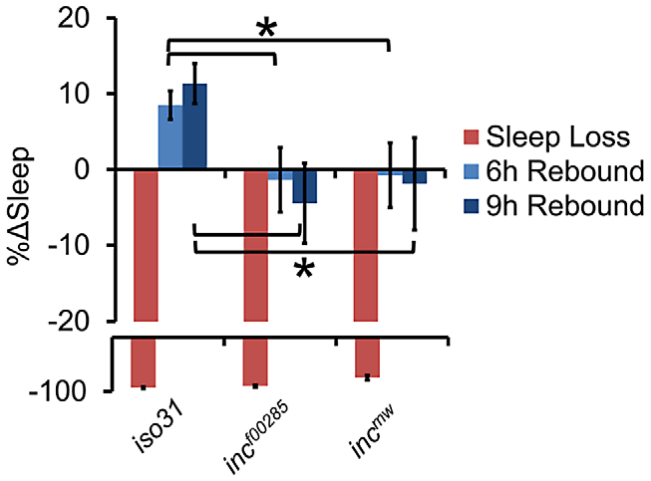
Flies lacking *inc* exhibit reduced behavioral sleep homeostasis. The graph shows the percent change in sleep (%Δsleep) after 12 h of mechanical sleep deprivation (red), 6 h of sleep recovery (light blue), and 9 h of sleep recovery (dark blue) in *inc^f00285^* (n = 45 females), *inc^mw^* (n = 68 females), and iso31 (n = 41 females) showing that the homeostatic response to sleep loss is disrupted in *inc* mutants. Error bars are SEM. * p<0.05 with Student's t test.

Although sleep duration and consolidation are important behavioral characteristics for determining the role a gene plays in sleep homeostasis, the gold standard is to analyze the homeostatic response to sleep loss, thus determining the role a gene plays in the sleep homeostat. Interestingly, whereas isogenic control flies had a significant increase in sleep after 12 hours of sleep deprivation by mechanical stimulation, we found that *inc* flies did not exhibit a detectable sleep rebound (Figure 4). Importantly, increasing the length of deprivation to 24 hours, which equalized the magnitude sleep loss in *inc* mutant flies to *iso31* 12 h sleep deprivation, still did not reveal a significant rebound in *inc* mutants (Figure S4).

### Cholinergic neurons likely mediate the effects of INC on sleep homeostasis

**Figure 5:**
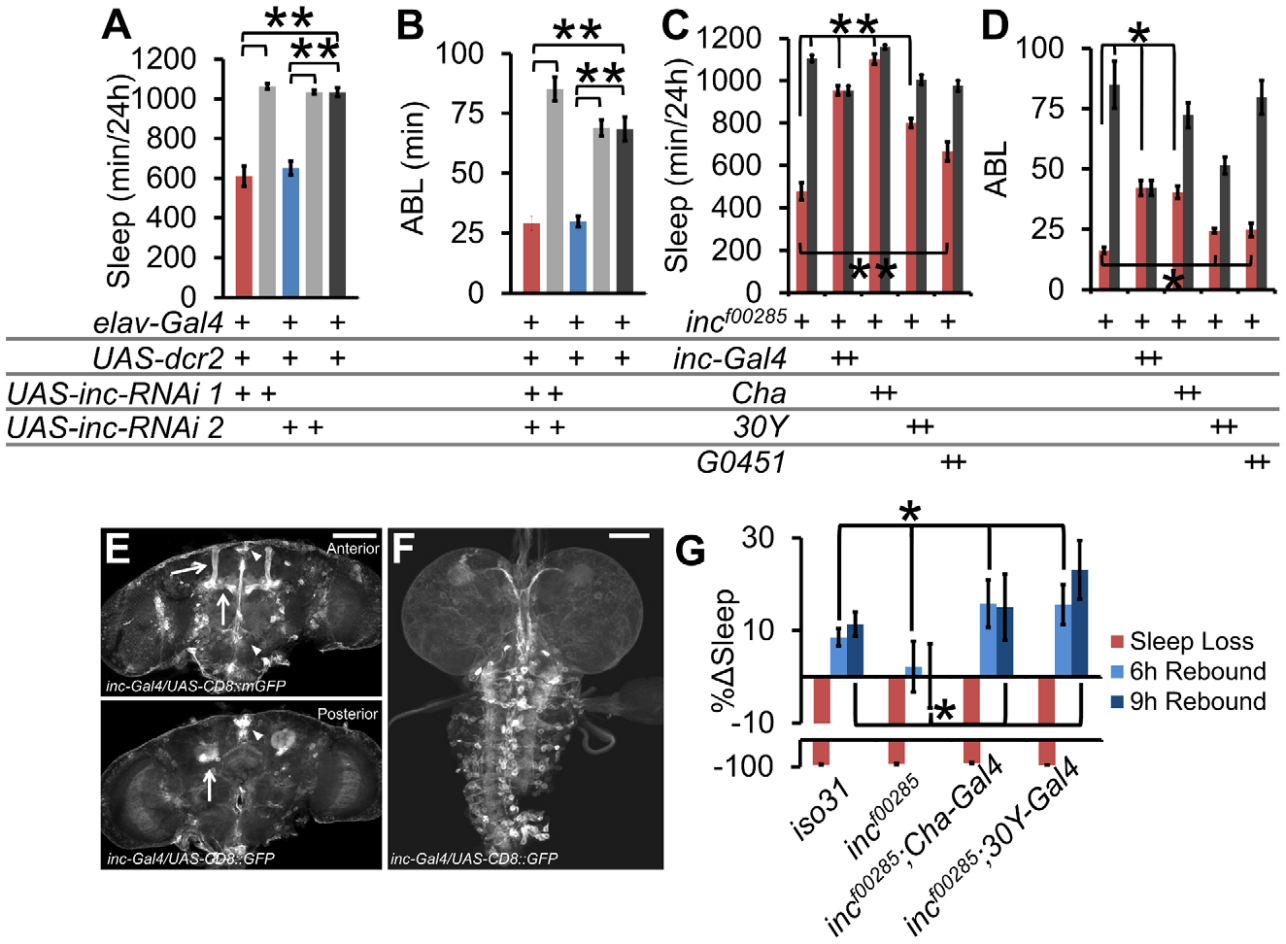
*inc* functional neuroanatomy. (A) Sleep duration and (B) average sleep bout length (ABL) in pan-neuronal knockdown of *inc* with 2 distinct *UAS-inc-RNAi* lines. (C) Sleep duration and (D) ABL in rescue of *inc^f00285^* (red) or wild type isogenic control (grey) with *inc-Gal4*, *Cha-Gal4*, *30Y-Gal4*, and *G0451-Gal4* (*inc^f00285^* is a *piggybac* insertion that contains a UAS element, n>30 male flies for all conditions). (E) *inc-Gal4* driving *UAS-CD8::GFP* in adult brain and (F) L3 wandering larval brain. Arrows identify the mushroom bodies (MB), triangle identifies the *pars intercerebralis* (PI). (G) Rescue of homeostatic sleep regulation in *inc^f00285^* (n = 45 females) by *Cha-Gal4* (n = 34 females), and *30Y-Gal4* (n = 36 females), as compared to *iso31* (n = 41 females). Graph shows the percent change in sleep (%Δsleep) after 12 h of mechanical sleep deprivation (red), 6 h of sleep recovery (light blue), and 9 h of sleep recovery (dark blue). Error bars are SEM. ** p<<0.001, * p<0.05 with Student's t test. Scale bars are 100 µm.

To test whether *inc* is required in neurons for proper sleep/wake regulation, we employed the *Gal4/UAS* system to knock down *inc* in all post-mitotic neurons with *elav-Gal4*. Using two distinct *UAS-inc-RNAi* transgenes targeting different parts of the *inc* transcript (Figure S2A) in concert with *UAS-dcr2* to enhance RNAi effects [22] we found that RNAi phenocopies the *inc* mutant sleep duration and consolidation phenotypes (Figure 5A–5B). We were unable to identify more-refined *Gal4* drivers that phenocopy the *inc* mutant sleep phenotype in combination with *UAS-inc-RNAi* alone.

Next we took advantage of the UAS element present in the *piggyBac* element in *inc^f00285^* (Figure S2A) to rescue *inc* phenotypes. We screened *Gal4s* with expression patterns in known sleep-regulatory regions of the brain, including the mushroom bodies (MB) (*247*, *30Y*, *c309*, *G0451*, *c305a*), the *pars intercerebralis* (PI; *50Y*, *c767*, *dilp2*), the ellipsoid bodies (EB; *c547*, *c305a*), circadian cells (*pdf*, *tim*), glia (*repo*), and a number of functional neuronal groups, such as dopaminergic neurons (Figure S5). In addition, we generated and tested flies in which the putative *inc* promoter (−2550…+340 bp relative to the transcription start site) drives *Gal4* expression. We find that both the cholinergic driver *Cha-Gal4* and one of the *inc-Gal4* lines provided rescue of the sleep duration phenotypes (Figure 5C–5D, Figure S5). Notably, the SH regulator *sleepless* (*sss*) also functions in *Cha-Gal4* neurons to regulate sleep [5]. Furthermore, the rescuing *inc-Gal4* line drove expression in known sleep regulatory loci including the MBs [23], [24], PI [11], [25], and fan-shaped body [26] as well as in the larval ventral nerve cord (Figure 5E–5F). Consistent with this pattern, we observed that 3 *Gal4* drivers that overlap in the MB gave partial rescue (Figure 5C–5D, Figures S5 and S6; *30Y*, *c309*, *G0451*); however, the more highly restricted MB driver *247-Gal4* did not rescue suggesting that additional neural loci and/or broader MB expression may be required.

We next sought to determine if *Gal4*s that rescue baseline sleep also rescue sleep homeostasis in *inc* mutants. We focused on *Cha-Gal4* and *30Y-Gal4* given their robust rescue and functional or regional expression specificity (Figure S6). We found that expression of *inc* with either *Cha-Gal4* or *30Y-Gal4* was sufficient to rescue sleep homeostasis defects (Figure 5G).

### CUL3 interacts with INC and regulates sleep homeostasis

**Figure 6:**
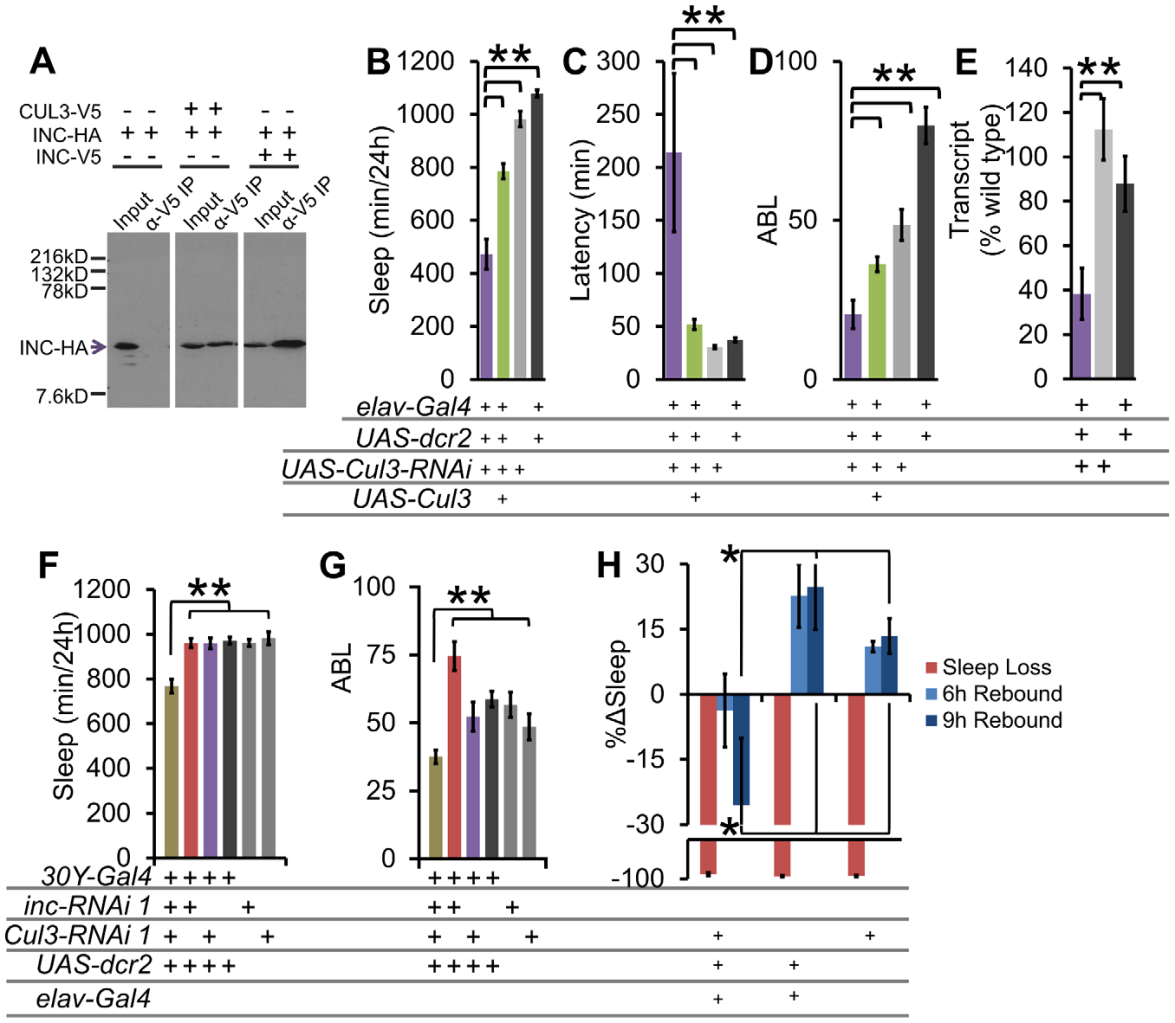
The INC-interactor CUL3 is necessary for proper sleep behavior. (A) Western blot shows results of an α-V5 immunoprecipitation from S2 cell supernatant transfected with *Cul3-V5*, *inc-HA*, and/or *inc-V5*, and blotted with α-HA to detect INC-HA. INC interacts with both CUL3 and itself under these conditions. Sleep duration (B), latency to sleep after lights-off (C), and average sleep bout length (ABL) (D) with *Cul3-RNAi* knockdown and *UAS-Cul3* rescue in post-mitotic neurons in males (n>26 male flies for all conditions). (E) Pan-neuronal knockdown with *Cul3-RNAi* results in reduced *Cul3* transcript levels as compared with heterozygous controls (n = 3×20 male heads/genotype). (F) Sleep duration and (G) ABL in *30Y-Gal4* knockdown of *inc* and *Cul3*. (H) Graph shows the percent change in sleep (%Δsleep) after 12 h of mechanical sleep deprivation (red), 6 h of sleep recovery (light blue), and 9 h of sleep recovery (dark blue) in *elav-Gal4;;UAS-dcr2/Cul3-RNAi* (n = 76 females), *elav-Gal4;;UAS-dcr2/+* (n = 31 females), and *Cul3-RNAi/+* (n = 54 females) showing that the homeostatic response to sleep loss is disrupted in flies lacking *Cul3*. Error bars are SEM. ** p<0.01, * p<0.05 with Student's t test.

The highly conserved mammalian homolog of INC, KCTD5, physically interacts with the E3 ubiquitin ligase CULLIN-3 (CUL3) and ubiquitin, consistent with a role as a substrate recognition adaptor for targeting ubiquitin-dependent degradation [16], [27]. In agreement with similar studies in *Drosophila* [21], we verified these interactions by co-immunoprecipitation of epitope-tagged CUL3 and INC proteins in S2 cells (Figure 6A). Furthermore, we found that INC could physically interact with itself, similar to findings reported for the mammalian homologue [16] (Figure 6A). To test if *inc* regulates sleep through *Cul3*, we analyzed pan-neuronal RNAi knockdown of *Cul3* (*Cul3* is an essential gene, and therefore there were no viable loss-of-function alleles to analyze). We found that 2 distinct insertions of an RNAi construct against *Cul3*, when driven in post-mitotic neurons, phenocopied the *inc* mutant phenotype, exhibiting reduced, poorly consolidated sleep, and increased latency to sleep. Furthermore, we found that the baseline sleep phenotypes could be partially rescued by co-expression of wild-type *Cul3* using *UAS-Cul3* (Figure 6B–6D, Figure S7A–S7C). Importantly, we observed reduced *Cul3* transcript levels with pan-neuronal *RNAi* knockdown (Figure 6E, Figure S7D).

To examine genetic interactions between *Cul3* and *inc*, we employed the *30Y-Gal4* driver in combination with RNAi. We found that whereas *30Y-Gal4* driven RNAi knockdown of either *inc* or *Cul3* alone was insufficient to affect sleep, knockdown of both simultaneously results in a significant synthetic decrease in sleep duration and consolidation (Figure 6F–6G), consistent with the hypothesis that each partially impairs the same pathway.

To determine if *Cul3-RNAi* displays a similar sleep homeostasis phenotype as *inc*, we examined the behavioral response of *Cul3-RNAi* flies to mechanically induced sleep deprivation. We did not detect any significant rebound in these *Cul3-RNAi* knockdown flies (Figure 6H). These results further support a role for the INC/CUL3 complex in sleep homeostasis.

### Developmental expression of *inc* and *Cul3* may contribute to adult sleep behavior

**Figure 7:**
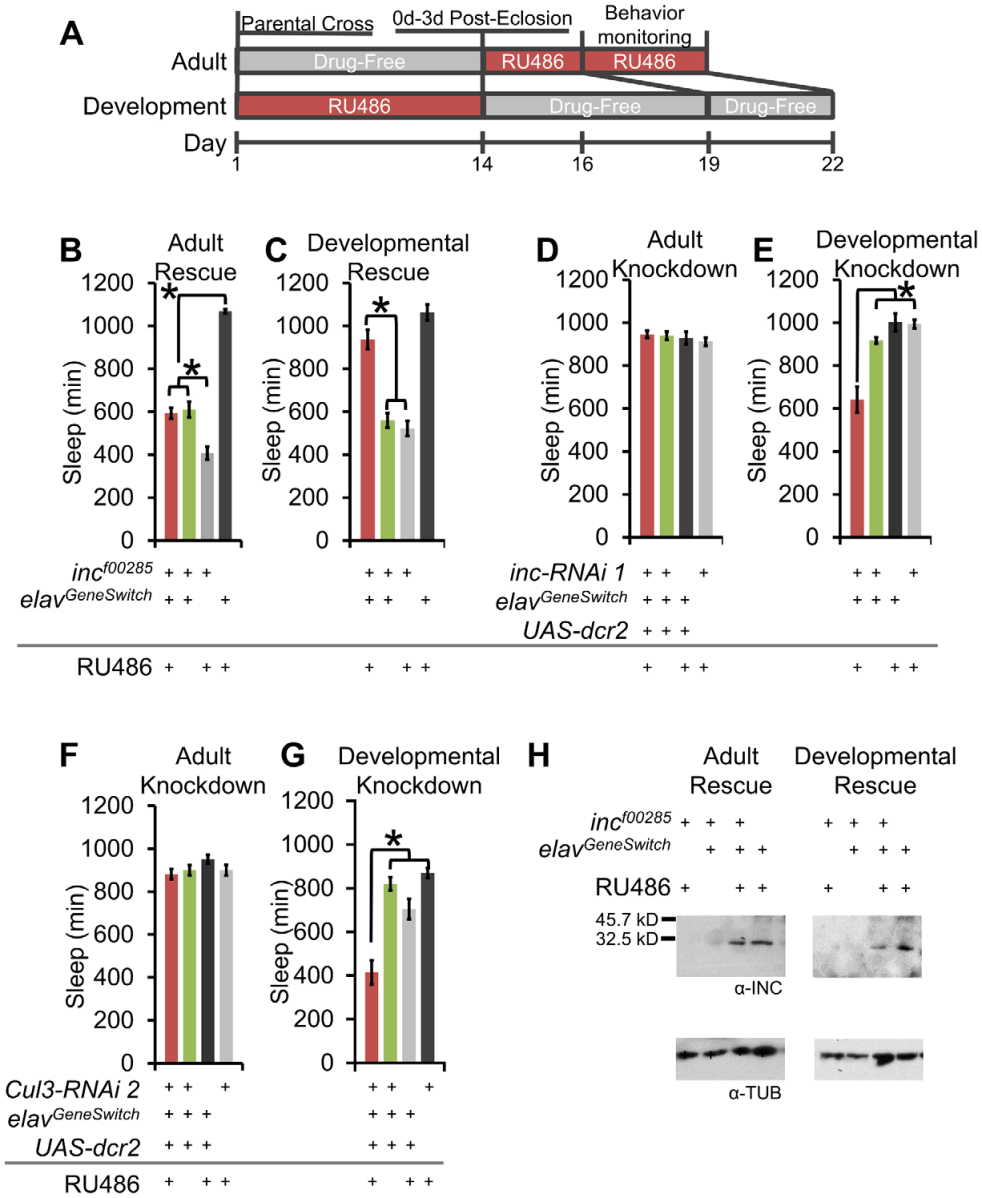
Developmental expression of *inc* and *Cul3* contributes to adult sleep behavior. (A) The schematic shows the time-course for administering RU486 for adult and developmental expression using *elav^GeneSwitch^*. (B–C) Graphs show sleep duration of *inc^f00285^* flies after pan-neuronal rescue starting 48 h prior to behavior (Adult Rescue) or from conception to eclosion ending 5 d before behavior (Developmental Rescue) with *elav^GeneSwitch^-Gal4* (n>37 male flies for all conditions). (D–E) Graphs show sleep duration after pan-neuronal RNAi knockdown of *inc* starting 48 h prior to behavior (Adult Knockdown) or from conception to eclosion ending 5 d before behavior (Developmental Knockdown) with *elav^GeneSwitch^-Gal4* (n>31 male flies for all conditions). (F–G) Graphs show sleep duration after pan-neuronal RNAi knockdown of *Cul3* starting 48 h prior to behavior (Adult Knockdown) or from conception to eclosion ending 5 d before behavior (Developmental Knockdown) with *elav^GeneSwitch^-Gal4* (n>36 male flies for all conditions). (H) Western blots show head INC levels for *elav^GeneSwitch^* driven rescue of *inc^f00285^* after adult RU486 exposure (3 d on RU486 or vehicle-laced after eclosion) or developmental RU486 exposure (5 d on drug-free food after eclosion) (INC levels with Adult v Dev rescue). α-TUBULIN (α-TUB) was used as a load control. RU486-induced *elav^GeneSwitch^* expression is stronger during development than during adult, to counteract this, developmental expression used 50 µM RU486 and adult expression used 500 µM. Error bars are SEM. * p<0.001 with Student's t test.

With few exceptions, and largely limited to overexpression [11], [14], prior discoveries of sleep mutants have not typically been accompanied by a direct assay to establish if effects are due to their function in development or in adulthood. To determine if *inc* and *Cul3* expression must be initiated developmentally or acutely in the adult to regulate sleep we employed the RU486-inducible pan-neuronal *Gal4* driver *elav^GeneSwitch^* [28]. To drive adult expression only, adult flies were placed on RU486-laced behavior food starting 48 h prior to monitoring sleep behavior. To initiate developmental expression, parent flies were mated on RU486-laced food, after which eclosed F1 progeny were removed to drug-free food for 5 days prior to monitoring behavior (Figure 7A). We find that *inc^f00285^;elav^GeneSwitch^* flies fed RU486- or vehicle-laced food after eclosion are indistinguishable for sleep (Figure 7B), whereas flies exposed to RU486, but not vehicle alone, during development exhibit rescue of sleep behavior (Figure 7C). Likewise, the short-sleep phenotypes observed with *inc-* and *Cul3-RNAi* knockdown were only present when driven during development, but not in adult flies (Figure 7D–7G). We next tested the effectiveness of *elav^GeneSwitch^*-driven rescue of *inc* by asking (1) is INC protein detectable in adult heads after adult only rescue and (2) is cessation of RU486 exposure for 5 d sufficient to remove residual INC protein? We found that *inc^f00285^;elav^GeneSwitch^* flies fed RU486-laced food as adults exhibit wild-type INC protein levels in their heads (Figure 7H). However, *inc^f00285^;elav^GeneSwitch^* flies exposed to RU486-laced food prior to eclosion retained some INC even after 5 d on RU486-free food (Figure 7H). These results suggest that INC has a long half-life, and raise the possibility that developmental transcription may contribute to adult protein levels. Taken together, these data demonstrate that *inc* and *Cul3* expression during development in post-mitotic neurons may contribute to adult sleep.

If *inc* and *Cul3* have developmental functions, we would predict morphological phenotypes, especially in sleep-regulatory regions. Whereas we observed no gross defects in the PI, clock neurons, or dopaminergic neurons (Figure S8), we did observe a low penetrant stochastic branching defect in the MB (Figure S9A–S9C). We labeled the MB with α-FASII immunofluorescence and 247dsRed to visualize the α/β, α′/β′, and γ lobes. Whereas all MB lobes were observable in 15 of 15 wild-type brains, a subset of *inc* mutant flies lacked either an α- or β-lobe (*inc^f00285^*: 10/50; *inc^mw^*: 12/38 brains; Figure S9B–S9C). These observed defects were non-symmetrical, such that only a single lobe was missing from a brain (i.e., the same lobe was present in the other hemisphere). Notably, *Cul3* has also been reported to play a role in MB branching [29]. We found that *Sh^mns^* and *DAT^fmn^* flies had normal MB morphology, arguing that reduced sleep or increased dopaminergic signaling alone is not the underlying cause of the morphological phenotype (Figure S9D–S9E). To determine if it was possible for these defects to be the underlying cause of the *inc* sleep phenotype we compared the morphological penetrance to the sleep behavior penetrance. Whereas 20–32% of *inc* mutants exhibited the MB morphological defect, >90% of *inc* mutant flies slept less than the shortest sleeping *iso31* fly (Figure S9F), and almost 75% of *inc* mutant flies exhibited less consolidated sleep than the most extreme *iso31* example (Figure S9G). Based on these findings the MB branching defect cannot be the sole cause of the sleep phenotypes.

### *inc* and *Cul3* regulate arousability

**Figure 8:**
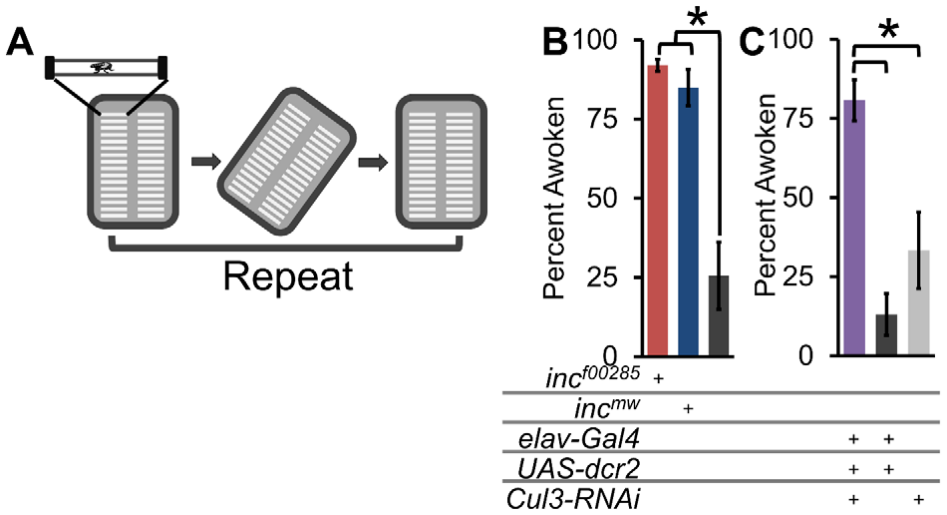
Flies lacking *inc* or *Cul3* are hyper-arousable. (A) Schematic shows arousal threshold paradigm. At ZT16, flies were rotated off horizontal and back, and the number of sleeping flies to wake up within 5 min were counted (see methods). (B) Graph shows arousal threshold (percent flies awoken by a rotational stimulus 2 h after lights-off) in *inc^f00285^* (red), *inc^mw^* (dark blue), and *iso31* (grey). (C) Graph shows arousal threshold in *elav-Gal4;Cul3-RNAi* (purple), *elav-Gal4* heterozygotes (dark grey), and *Cul3-RNAi* heterozygotes (light grey). n>55 sleeping male flies for all genotypes. Error bars are SEM. * p<0.01.

To further elucidate the underlying mechanism of the *inc/Cul3* phenotype, we next asked whether *inc* flies had difficulty maintaining and initiating sleep because they were hyper-arousable. To test arousability, we used a mechanical apparatus to rotate the DAM monitors, and therefore the glass capillary tubes that housed the flies, off horizontal at ZT16 and examined the waking response of flies that were asleep prior to the stimulus (Figure 8A, see methods). Controlling for flies that spontaneously awoke in the absence of a stimulus, we found that whereas roughly 25% of sleeping wild-type flies woke up in response to this rotational stimulus, >85% of *inc^f00285^* and *inc^mw^* flies woke up, arguing that *inc* mutants indeed are hyper-arousable (Figure 8B). In addition, we also examined arousability in flies expressing *Cul3*-*RNAi* pan-neuronally and observed similar results with >80% of flies responding to the stimulus (Figure 8C). This makes *inc* mutants and *Cul3*-RNAi flies distinct from *Sh^mns^* and *CnA* knockdown flies, which exhibit short-sleep phenotypes in the absence of hyper-arousability [15], [30].

### *inc* effects on sleep require dopamine

**Figure 9:**
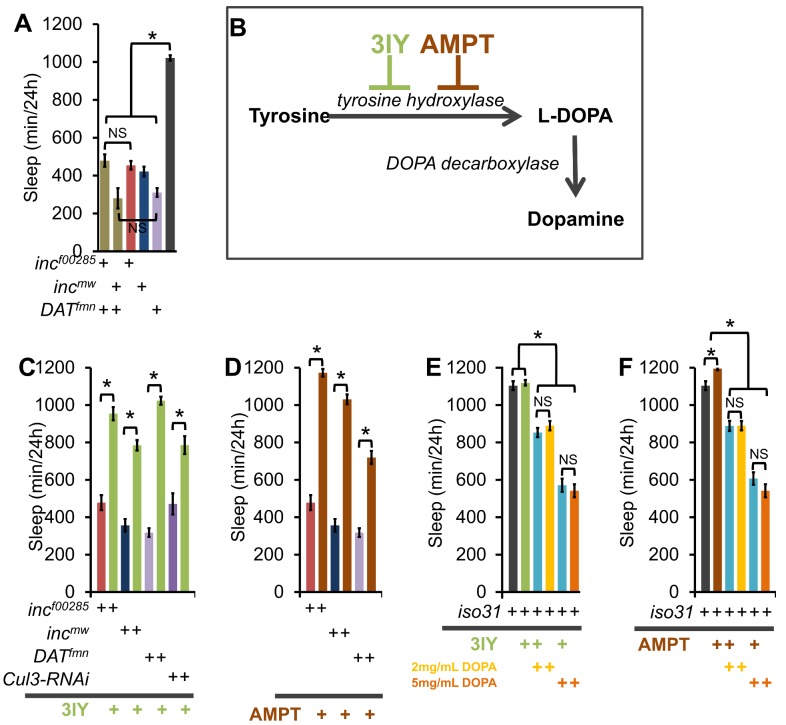
*inc* effects on sleep require a dopamine sleep-regulatory pathway. (A) Graph shows sleep duration in *inc^f00285^;DAT^fmn^* (tan), *inc^mw^;DAT^fmn^* (tan), *inc^f00285^* (red), *inc^mw^* (dark blue), *DAT^fmn^* (light purple), and *iso31* (grey) males showing a non-additive effect on sleep duration between *inc* and *DAT^fmn^* (n>38 males for all genotypes). (B) Schematic shows the dopamine synthesis pathway. *3-iodo-tyrosine* (3IY) and *α-methyl-p-tyrosine methyl ester* (AMPT) are inhibitors of *tyrosine hydroxylase* (TH). (C–D) Graphs show sleep duration in *inc^f00285^* (red), *inc^mw^* (dark blue), *DAT^fmn^* (light purple), and *elav-Gal4;UAS-Cul3-RNAi/UAS-dcr2* (*Cul3-RNAi*, dark purple) after consumption of food laced with TH antagonists 2 mg/mL 3IY (**C;** green) or 1 mg/mL AMPT (D; brown; n≥31 males for all conditions). (E–F) Graphs show effects of 2 mg/mL 3IY (green), 1 mg/mL AMPT (brown), 2 mg/mL L-DOPA (yellow), and 5 mg/mL L-DOPA (orange) on sleep duration in *iso31* flies. L-DOPA is the product of TH, and the precursor to dopamine. A non-significant effect of 3IY or AMPT on the reduction in sleep caused by L-DOPA (blue) demonstrates pathway specificity for these TH inhibitors (n≥31 males for all conditions). Error bars are SEM. * p<0.001 with Student's t test.

Dopaminergic signaling is a key regulator of arousal in both flies and mammals [7]–[10], [31], [32]. To determine if *inc* functions in a dopaminergic arousal pathway we first took a genetic approach and asked if the sleep duration phenotype in *inc* mutants was additive with the dopamine transporter mutant *DAT^fmn^*. We found that *inc^f00285^;DAT^fmn^* and *inc^mw^;DAT^fmn^* flies did not sleep significantly less than single mutants (Figure 9A), in support of our hypothesis that *inc* and *DAT* operate in the same arousal pathway.

To test the specificity of *inc* function we took a pharmacological approach to modulate two known arousal pathways. Flies were fed (1) L-DOPA to increase dopaminergic arousal or (2) carbamazepine (CBZ), an antagonist of the *GABA* receptor *Rdl* previously demonstrated to increase arousal by modulation of the PDF cells [33]–[35]. We found that, whereas wild-type *iso31* and *inc* mutants exhibit robust reductions in sleep with CBZ (Figure S10A), *inc* mutants were resistant to the sleep effects of L-DOPA (Figure S10B) arguing that the *inc* effects are specific to a dopaminergic, but not the *Rdl*, arousal pathway. The CBZ data further indicate that there is not a “floor” effect preventing further reductions in *inc* sleep.

We next pharmacologically examined the dopamine-dependence of *inc* and *Cul3* sleep phenotypes. Flies were fed one of two inhibitors of the rate limiting step in dopamine synthesis, *tyrosine hydroxylase* (TH): (1) 3IY (*3-iodo-tyrosine*) [36], or (2) AMPT (*α-methyl-p-tyrosine methyl ester*, Figure 9B) [37]. Inhibition of dopamine synthesis with either drug suppressed the *inc* mutant and *Cul3*-*RNAi* short-sleep phenotypes, as well as that of *DAT^fmn^*, which is thought to increase arousal through increased dopaminergic signaling [9] (Figure 9C–9D). One possibility is that 3IY and AMPT act non-specifically to increase sleep; therefore, we next sought to determine the specificity of these drugs by restoring L-DOPA, the enzymatic product of TH (Figure 9B). We found that wild-type *iso31* flies fed L-DOPA alone exhibited a dose-dependent decrease in sleep; furthermore, 3IY and AMPT did not suppress the L-DOPA effect, as expected if they operate upstream of L-DOPA (Figure 9E–9F). Importantly, we also observed decreased head dopamine levels after 3IY consumption, and increased levels after L-DOPA consumption, biochemically verifying drug mechanism/efficacy (Figure S11). Interestingly, *inc* phenotypes were not rescued by expression in dopaminergic neurons using a *tyrosine hydroxylase-Gal4* (*TH-Gal4*) or a *Dopa decarboxylase-Gal4* (*Ddc-Gal4*) (Figure S5); furthermore, TH protein levels were not altered in *inc* mutant brains (Figure S11C), suggesting that *inc* does not control sleep via its function in dopaminergic neurons.

**Figure 10:**
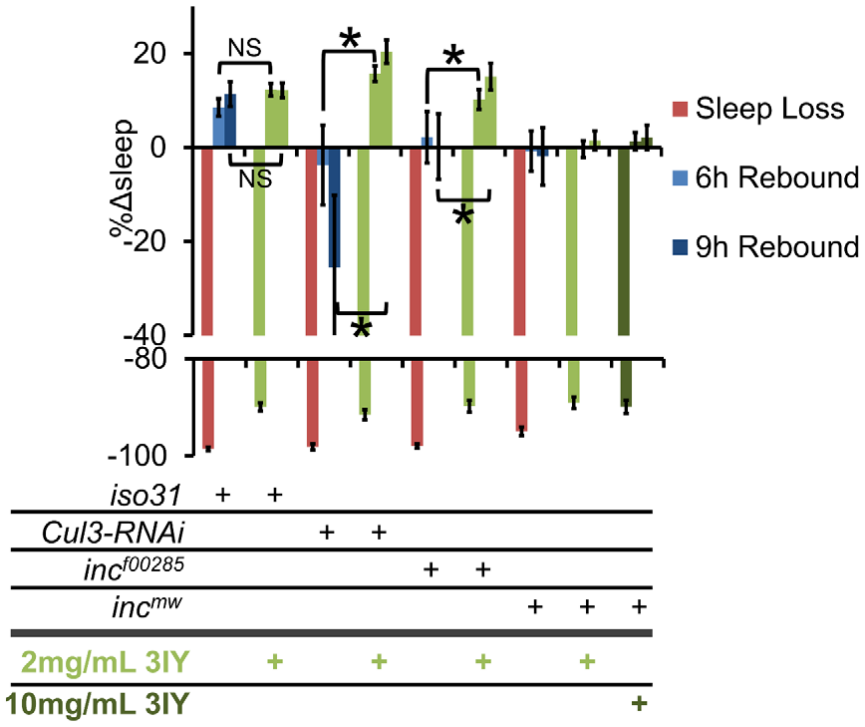
3IY rescues sleep homeostasis defects in *Cul3-RNAi* and *inc^f00285^* but not *inc^mw^*. The graph shows the percent change in sleep (%Δsleep) after 12 h of mechanical sleep deprivation (red), 6 h of sleep recovery (light blue), and 9 h of sleep recovery (dark blue) in *iso31*, *elav-Gal4;Cul3-RNAi/UAS-dcr2 (Cul3-RNAi)*, *inc^f00285^*, and *inc^mw^* with 2 mg/mL 3IY (light green), 10 mg/mL 3IY (dark green) or no 3IY. n≥41 females for all conditions. * p<0.05. Error bars are SEM.

Given that *Cul3* and *inc* affect sleep homeostasis and dopaminergic arousal pathways, we next sought to determine if these phenotypes are linked. We found that wild-type flies fed 3IY displayed intact sleep homeostasis (Figure 10). On the other hand, 3IY consumption restored sleep homeostasis in *Cul3*-*RNAi*, indicating the dopamine dependence of the homeostatic defect in these flies. We also found that 3IY could restore rebound sleep in *inc^f00285^* but not in *inc^mw^* (Figure 10). While the molecular lesion differs between the two *inc* alleles, we are not aware of how this might explain the different drug responses between these two mutants. Although we did test a 5-fold higher concentration of 3IY than that sufficient to rescue *Cul3-RNAi* and *inc^f00285^* and found that it was still insufficient to rescue *inc^mw^* (Figure 10), we cannot rule out a trivial explanation for the negative results in *inc^mw^* such as insufficient drug uptake/dopamine suppression. Nonetheless, the positive results with *Cul3* and *inc^f00285^* support a model in which the homeostatic defect in these flies depends on dopamine. We are not aware of another example of pharmacological rescue of sleep rebound, at least in *Drosophila*. Taken as a whole, these results suggest that *inc* functions in a group of cholinergic neurons, as defined by *Cha-Gal4* and *30Y-Gal4*, and that in its absence excess dopaminergic signaling underlies the resulting sleep phenotype.

## Discussion

Our detailed phenotypic analyses of *inc* and *Cul3* reveal novel insights into the molecular basis of sleep homeostasis and dopaminergic arousal pathways. *inc* mutants display one of the strongest baseline and homeostatic sleep phenotypes thus far observed. Furthermore, we find that the reduced sleep phenotype depends largely on a single neurotransmitter, dopamine, establishing a transmitter basis to *inc* function.

### Widespread modulatory effects of genetic background on sleep

Using genetic backcrossing to an isogenic (*iso31*) strain, we made the striking observation that the vast majority of the mutants (39/45, nearly 90%) identified in our primary genetic screen did not have a significant phenotype after backcrossing, indicating a remarkably pervasive role for genetic background in mediating sleep phenotypes in a variety of mutant strains. There are two main possibilities for how genetic background influences sleep phenotypes: (1) the tested allele indeed affects sleep; however, there are suppressors of this phenotype present in the *iso31* background but absent from the original background. (2) The sleep phenotype is not due to the transposon insertion but instead is caused by one or more flanking mutations present in the original mutant background but absent from the *iso31* background. Isolated examples of (1) have been observed in the case of *Sh* and mutants of the *Sh* regulatory subunit *Hyperkinetic* [6], [15] as well as *Crc* and *Sema-5c* effects on olfactory, startle, and sleep behavior [38] and of (2) in the discovery of *DAT^fmn^* mutants in the background of a *timeless* mutant strain [9]. These are consistent with observations in *C. elegans* indicating the limitations of backcrossing for removing flanking mutations [39] and in *Drosophila* indicating the widespread presence of background mutations that can suppress mutant-induced behavioral phenotypes [38]. Our experience with *RhoGDI^EY02738^* suggests that scenario 2 may be more common than previously thought (Figure S1C). Nonetheless, the sheer number of examples observed here indicates that the presence of genetic variation at sleep regulatory loci among laboratory stocks is both prevalent and perhaps even sufficiently important to mask or induce significant sleep phenotypes. Moreover, in the case of *Sh* a single outcross was sufficient to unmask the short sleep phenotype. Indeed, we assumed that if we outcrossed mutant alleles to a deficiency strain this would effectively remove the influence of accumulated recessive mutations that flank the allele. However, our backcrossing data indicates that this strategy did not remove those concerns, suggesting that background variants may exert dominant effects (see also [38]). Practically, our experience suggests that outcrossing to deletion stocks alone may not be sufficient to verify the function of a genetic locus in sleep. Overall, this observation has important implications for the role of genetic modifiers in sleep, the conduct and design of sleep genetic screens, and for the interpretation of sleep and other behavioral mutant phenotypes in general. While backcrossing can remove flanking genetic variants that may contribute to an observed phenotype, alone it is not sufficient to definitively establish genotype-phenotype causation.

### *inc* and *Cul3* are key regulators of sleep homeostasis in *Drosophila*

Despite the large modulatory effect of genetic background, we were able to observe persistent phenotypes with *inc*, which showed the most robust and reproducible sleep phenotypes, in particular demonstrating an important role in the homeostatic regulation of sleep. Several independent lines of evidence support the role of *inc* in sleep homeostasis. First, 2 *inc* alleles (*inc^f00285^* and *inc^mw^*) were backcrossed for 5 generations into an isogenic background and retained their short sleep and suppressed sleep homeostasis phenotypes, each among the strongest observed, as compared to isogenic control lines. Second, we rescued *inc^f00285^* in 2 distinct ways: (1) with genomic duplications encompassing the gene but not those that do not include the gene, and (2) using the *GAL4/UAS* system, the latter rescuing both baseline and homeostatic phenotypes. Third, we demonstrated failure to complement with a deletion removing the *inc* genomic locus, or *inc* transheterozygotes. Fourth, we demonstrated that two independent RNAi lines that target two different regions of *inc* phenocopy the *inc* mutant phenotype.

In addition, we provided evidence that the INC-interacting protein, the E3 ubiquitin ligase CUL3, functions to regulate sleep levels suggesting that *inc* links protein turnover to sleep homeostasis. Two independent inserts of a *Cul3-RNAi* line that effectively suppress *Cul3* mRNA levels resulted in reduced sleep, and induction of a wild-type *Cul3* transgene could rescue these phenotypes. We verified the CUL3/INC interaction in S2 cells and observed synthetic genetic interactions between *Cul3* and *inc* using RNAi, consistent with the model that they operate together to affect sleep.

A core concept in understanding sleep behavior is its homeostatic regulation, i.e., the observation that the drive to sleep reflects the duration of prior wakefulness. Sleep homeostasis typically is measured by enforcing wakefulness/depriving sleep for a defined period and assaying the increase in subsequent rebound sleep. Importantly, we demonstrated that both *inc* and *Cul3* have robust effects on sleep homeostasis where reduced *inc* or *Cul3* was accompanied by suppressed or absent sleep rebound under our conditions. These results suggest that *inc* and *Cul3*, and by extension, protein degradation, are important for the accumulation of sleep need during wake and/or dissipation of sleep need after deprivation.

### Expression of *inc* and *Cul3* during development in post-mitotic neurons may contribute to adult sleep

For the large majority of sleep mutants that have been described, assessment of developmental and adult contributions has not formally been addressed, raising questions regarding their precise function in sleep. Here we provided evidence that *inc* induction or *Cul3-RNAi* knockdown during development, but not exclusively during adulthood, could rescue (in the case of induction) or phenocopy (in the case of knockdown) their respective mutant/RNAi phenotypes. The *Cul3* results are consistent with an established role for *Cul3* in dendritic and axonal arborization, in which dendritic and axonal arborization are reduced in *Cul3* mutants [29], [40]. Our data also revealed a stochastic branching defect in MB neurons in 26% of *inc* mutants, in which they lack a single α- or β-lobe. Based on the incomplete penetrance of this morphological defect, it cannot explain the sleep behavior phenotype; however, it may be reflective of other morphological phenotypes that are causative for behavior. Alternatively, the necessity for developmental expression of *Cul3* and *inc* may be for the appropriate processing, maturation and/or localization of these proteins in the adult. The apparent long half-life/persistence of this protein after induction only during development is consistent with the possibility that developmentally expressed transcription is important for adult protein expression and function. Regardless, it will be of interest to examine the relative adult and developmental requirements of other sleep mutants.

### *inc* and *Cul3* function in a dopaminergic arousal pathway

We found that the reduced sleep phenotype depends largely on a single neurotransmitter, dopamine, establishing a transmitter basis to *inc/Cul3* function. Dopaminergic signaling is a key regulator of sleep/wake behavior. In humans, sleep deprivation has been associated with increased brain levels of dopamine [41]. Treatment of Parkinson's disease with L-DOPA can alleviate daytime sleepiness, or in the extreme result in insomnia [42]–[45]. In *Drosophila*, genetic loss or pharmacological inhibition of *tyrosine hydroxylase* increases sleep [46]–[48]. Furthermore, flies that lack a functional copy of *DopR* exhibit increased sleep and general arousal defects, including reduced arousing effects of caffeine [8], [49]. Conversely, in *DAT^fmn^* flies, or flies fed dopamine-enhancing methamphetamine, sleep levels are severely reduced [9], [48]. Dopamine arousal effects are modulated by light [50]. Moreover, sleep deprivation induced reductions in learning can be suppressed by enhancing dopaminergic signaling [51]. Other than dopamine receptors and *DAT*, members of the dopaminergic arousal pathway remain largely unknown.

We report here that *inc* and *Cul3* function in the dopaminergic arousal pathway. First, *inc* mutants, *Cul3*-*RNAi*, and *DAT^fmn^* all showed robust sleep duration and consolidation phenotypes. Second, all three groups were hyper-arousable to mechanical stimuli. Third, disruption of *inc*, *Cul3*, and *DAT* all exhibited suppressed or absent homeostatic responses to sleep deprivation. Fourth, the short-sleep phenotypes of *inc* and *DAT^fmn^* were non-additive in double mutants. Fifth, while wild-type flies exhibited reduced sleep when fed the dopamine precursor L-DOPA, *inc* mutants were resistant to these effects, but not the arousing effects of the *Rdl* antagonist CBZ. Finally, the sleep duration phenotypes in flies with disrupted *inc*, *Cul3*, and *DAT* could be suppressed by pharmacologically inhibiting dopamine synthesis with 3IY or AMPT, linking short sleep to excess dopamine function. Importantly, we demonstrated that inhibition of dopamine synthesis via *tyrosine hydroxylase* inhibition does not affect L-DOPA-induced sleep reductions. We also observed that 3IY could restore sleep homeostasis to *Cul3-RNAi*. Similar 3IY effects on homeostasis were only observed in one of the two *inc* alleles. Nonetheless, these studies do further link dopamine signaling to sleep homeostasis. To our knowledge *inc* and *Cul3* are the first genes that are not known dopamine receptors reported to function in the dopaminergic arousal pathway, further reinforcing the pivotal role of dopamine in sleep homeostasis.

Our data suggests *Cul3/inc* function to regulate dopaminergic signaling downstream of dopamine. *inc* phenotypes did not map to dopaminergic neurons nor were we able to identify consistent changes in global dopamine levels among *Cul3-RNAi* and *inc* mutants (data not shown). Thus, *Cul3/inc* may be involved in active turnover of dopamine receptors or their effectors in neurons defined by *Cha-GAL4* and *30Y-GAL4*. We examined double mutants of *inc* and a major dopamine receptor involved in arousal in *Drosophila*, *DopR*, and failed to observe suppression of *inc* baseline phenotypes; moreover, we found that *DopR* mutant flies were responsive to 3IY consumption (i.e. exhibit increased sleep; data not shown), suggesting that additional dopamine receptors function in *Cul3/inc*-based dopamine arousal. *Drosophila* has 2 other dopamine receptors and we have observed partial suppression of *inc* with *DopR* and *DopR2* RNAi (data not shown), suggesting that multiple dopamine receptors may contribute to these effects. Alternatively, *Cul3/inc* may be important for protein turnover of other homeostatically regulated components. For example, extensive and dose-dependent changes in synaptic protein expression throughout the brain with sleep deprivation and recovery [52]–[54] may depend on *Cul3/inc*-dependent turnover of these proteins during sleep.

Interestingly, *Cul3* has also been linked to sleep behavior via a candidate gene for Restless Leg Syndrome (RLS) and BTB gene, *BTBD9* [55]. Unlike our studies, disruption of the *Drosophila BTBD9* is not associated with reduced sleep, a reduced level of waking activity, nor elevated dopaminergic signaling. In addition, the phenotypes map in part to dopaminergic neurons in the case of *BTBD9* rather than cholinergic neurons for *inc*. Thus, *Cul3/inc* likely represents a distinct pathway regulating sleep. Nonetheless, these studies further highlight the importance of *Cul3*/BTB adaptor pathways in sleep regulation in both *Drosophila* and humans. Future work will be required to identify the dopamine and sleep-relevant ubiquitination target(s) of *inc* and *Cul3*.

## Materials and Methods

### Flies

Flies were raised on cornmeal-yeast-agar food at 25°C, 12 h∶12 h Light∶Dark. Alleles and deficiencies for the reverse-genetics screen were acquired from the *Drosophila* Stock Centers based in Bloomington, Kyoto, Harvard, and Szeged. The deficiencies were from the isogenic collection created by DrosDel. Stocks of particular interest: *inc*-spanning duplications (Bloomington #6021 [Dp(1;Y)Sz280], 33872 [Dp(1;Y)BSC308], 33871 [Dp(1;Y)BSC307]), non-*inc*-spanning (#33875 [Dp(1;Y)BSC311]), *inc*-spanning deficiency (Bloomington #934 [Df(1)S39]), *inc*-RNAi (Vienna Drosophila RNAi Center #18226, #108816), *Cul3-*RNAi (National Institute of Genetics – Kyoto #11861R-1, #11861R-2), UAS-*Cul3* (Bloomington #9936), *Cdk4-*spanning deficiency (Bloomington #9213 [Df(2R)ED3181]), *mXr-*spanning deficiency (Bloomington #9276 [Df(2R)ED1742]), *CG9135-*spanning deficiency (Bloomington #9186 [Df(2L)ED353]).

The following Gal4s were used: *50Y*, *c929*, *5HT7*, *5HT1a*, *ple*, *Ddc*, *DopR*, *DopR2*, *Hdc*, *Tdc*, *vGlut*, *repo*, *Cha*, *elav* (Bloomington #30820, 25373, 23066, 27807, 27820, 8848, 7010, 24743, 19491, 25260, 9313, 26160, 7415, 6793, 8765), *247*, *30Y*, *c309*, *c767*, *c547*, *c305a* [24], *pdf* [34], *tim* [56], *Trh* [57], *G0451* [58], *dilp2* [59], *Gad* [60], *elav^GeneSwitch^* [28].

*inc*-Gal4 was created using 3 Kb upstream of the *inc* ATG start site (−2550–+340 bp relative to the transcription start site) from an *inc* genomic BAC (CHORI: CH223-4018). The promoter region was inserted into pPTG4, and the *inc*-Gal4 vector was injected into embryos by BestGene Inc.

*inc^mw^* was created by cloning 4197 bp upstream of the *inc* stop codon (left arm) and 4052 bp downstream and including the stop codon (right arm) into pw25 (DGRC 1166), flanking a *miniwhite* gene. Constructs were injected into embryos by BestGene Inc. To knock in the *miniwhite*, the fragment with *inc* flanking regions was mobilized by hsFLP, digested *in vivo* with Sce-I (Bloomington #6934), and candidate knock-in flies were screened behaviorally and molecularly by PCR.

### Conditional expression

For adult-specific expression with *elav^GeneSwitch^*, flies within 3 d after eclosion were placed on behavior food laced with 500 µM RU486 (Sigma) or vehicle alone (4% ethanol final concentration) for 48 h prior to behavior monitoring, and then monitored for 3 d in behavior. For developmental expression, parental flies were crossed on normal cornmeal-based food laced with 50 µM RU486 or vehicle alone (0.4% ethanol final concentration). Within 3 d after eclosion, F1 progeny were moved to drug-free food for 5 d prior to behavior monitoring, and then monitored for 3 d in behavior.

### Genetic screen

To identify new genetic regulators of sleep we screened genes with sleep/wake-regulated expression [3], [17], genes with circadian expression patterns [3], [17]–[19], enriched in the MB [61], and genes involved in neuronal and intracellular signaling (flybase.com). We screened 1297 alleles covering 1015 genes. In each case a previously existing allele was tested over a deficiency (Df) from the isogenic DrosDel collection [20], and allele/Df combinations shifted ≥2SD from the population mean for sleep duration and/or average sleep bout length in males were considered hits. In the case of X-linked genes, allele virgins were crossed to X-linked deficiency males and the F1 allele/Y males were tested. Whenever possible we tested proven loss-of-function alleles, followed by mutations that affect in descending order of preference: exonic regions, 5′untranslated region, 3′untranslated region, intronic regions, promoter regions.

### Quantitative PCR

Flies 4–8d post-eclosion were frozen on dry ice, heads were removed by dry ice cold vortexing and isolated on frozen sieves. RNA was isolated from 20 heads/sample with Trizol (Invitrogen). qPCR was performed using a QuantiTect SYBR Green PCR Kit (Qiagen).

### Behavior

Sleep behavior was analyzed using the Drosophila Activity Monitor system (Trikinetics), and processed with a custom written Excel macro [62]. Flies 2–5d post-eclosion were individually loaded into 5×65 mm glass capillary tubes with a 5% sucrose 2% agar food source and analyzed for 5 d 25°C 12 h∶12 h Light∶Dark. Sleep was defined as ≥5 min inactivity (zero infrared beam crossings).

To determine lifespan, flies were maintained in DAM monitors as described above and transferred to fresh behavior tubes every 7 d.

### Sleep deprivation

Sleep deprivation was performed as described previously [24]. Briefly, activity monitors were placed in an apparatus that rotates and jostles the flies at varying intervals. Flies age-matched within 24 h were loaded into behavior 2 d after eclosion and allowed at least 36 h to acclimate followed by 24 h without sleep deprivation to determine 24 h baseline sleep. For behavioral analyses flies were deprived ZT11–ZT23 for 12 h deprivation and ZT0-ZT0 for 24 h deprivation. Non-deprived controls were handled similarly to deprived flies, in a separate incubator from the sleep deprivation apparatus. We confirmed behaviorally that the flies lost ≥90% of their sleep with this protocol. To determine Δsleep, baseline sleep was subtracted from sleep obtained during the recovery period for individual flies from both sleep-deprived and non-deprived populations then non-deprived Δsleep was subtracted from sleep-deprived Δsleep.

### Arousability

Flies were loaded into the same apparatus as for sleep deprivation (see above Methods section), and given 1 day to acclimate. On the second night flies were stimulated by mechanically rotating the DAM monitors 36° off horizontal and back 10 times for *inc* experiments and 18° 2 times for *Cul3* experiments at ZT16, and the number of sleeping flies to wake up within 5 min of the stimulus was determined. We took into account the number of flies that would wake spontaneously by determining the number of flies to wake up at ZT15:50 and normalizing the percent awoken with the following formula:
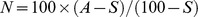


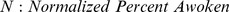


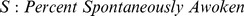


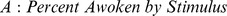



### Dopamine drug treatment

Flies were raised on cornmeal-yeast-agar food and presented with drug-labeled food throughout the behavior experiment. 3IY, AMPT, and L-DOPA were dissolved in 5% sucrose 2% agar behavior food as the sole food-source during the behavior experiment. For 3IY- and AMPT-only experiments, flies were presented with drug-laced food for 12–16 h before monitoring behavior for 2 d. For L-DOPA experiments, flies were presented with drug-laced food for 12–16 h before monitoring behavior for 1 d (after 48 h on L-DOPA the flies become unhealthy).

### Immunostaining and quantification

The following antibodies were used in this study: ms-α-PDF (DSHB; 1∶100), rab-α-tyrosine hydroxylase [63] (1∶1000), ms-α-FASII (DSHB: 1∶50), gt-α-ms-Alexa488, gt-α-rab-Alexa488 (Invitrogen; 1∶500). Flies were dissected in 1% TritonX-100, 3.7% formaldehyde in PBS, fixed for 1 h post-dissection in 3.7% formaldehyde in PBS, and permeablized in 0.3% TritonX-100 in PBS overnight. Brains were incubated with all antibodies in 0.3% TritonX-100, 7% goat normal serum in PBS overnight. Wash steps were with 0.3% TritonX-100 in PBS.

### Coimmunoprecipitations and Western blotting

Expression constructs were made using *inc* cDNA (DGRC #GM03763) and *Cul3* cDNA (DGRC #LD10516). V5-tagged constructs were made by cloning the coding region into pAc5.1-V5/His (Invitrogen). HA-tagged constructs were made using a modified version of pAc5.1-V5/His, in which the coding region for V5/His was replaced with HA. Transfections were done on *Drosophila* S2 cells using Effectene reagent (Qiagen). 24 h after transfection, cells were lysed with T150 lysis buffer (25 mM Tris-Cl pH 7.5, 150 mM NaCl, 10% glycerol, 1 mM EDTA, 1 mM DTT, 0.5% NP-40, 1 mM PMSF), supernatant was mixed with α-V5 agarose beads (Sigma) for 1.5 h at 4°C, after washing beads were boiled with SDS loading buffer, and eluted sample was run on 10% acrylamide gels. Hybond membranes (GE Lifesciences) were blotted with α-HA (1∶2500, Roche), and developed with ECL Plus (GE Lifesciences).

For INC head western blots, 30 flies/sample were flash frozen on dry ice at ZT6, and heads were homogenized in 30 µL lysis buffer (20 mM HEPES [pH 7.5], 100 mM KCl, 10 mM EDTA, 50 mM NaCl, 0.1% Triton X-100, 10% glycerol). Samples were run on 15% acrylamide gels. Hybond membranes (GE Lifesciences) were blotted with α-INC (1∶2000 [21]), and developed with ECL Prime (GE Lifesciences).

### Dopamine levels

20 age-matched male flies were presented with drug-free behavior food, or food laced with 2 mg/mL 3IY, 2 mg/mL L-DOPA, or 5 mg/mL L-DOPA for 2 d under 12 h light∶12 h dark conditions. They were then frozen on dry ice at ZT6, heads were removed by dry ice cold vortexing and isolated on frozen sieves. Dopamine levels were determined by HPLC by Dr. Raymond F Johnson at the Vanderbilt University Neurochemistry Core Lab.

### Statistics

To compare quantifiable groups with normal distributions (as determined by the Shapiro-Wilk Test) we used the two-tailed Student's t test. To compare sleep bout lengths, which are not normally distributed, we used the Mann-Whitney U Test. p<0.05 was considered statistically significant.

## Supporting Information

Figure S1 Genetic background modulates sleep phenotypes. (A) Sleep duration in *mXr^DG17503^/Df(2R)ED1742* in the original genetic background (tan, n = 20 males) as compared to the *iso31* background (blue, n = 10 males) and the screen population average (grey, >4000 males). (B) Average sleep bout length (ABL) in *CG9135^f03307^/Df(2L)ED353* in the original genetic background (tan, n = 20 males) as compared to the *iso31* background (blue, n = 10 males) and the screen population average (grey, >4000 males). (C) Sleep duration in *RhoGDI^EY02738^/Df(3L)ED4858* in the original genetic background (tan, n = 20 males) as compared to the *iso31* background (blue, n = 10 males), the screen population average (grey, >4000 males) and a precise excision revertant (*RhoGDI^EY02738-rev^*, light grey, n = 10 males). Error bars are SEM. * p<0.001.
(PDF)

Figure S2 *inc^f00285^* sleep phenotypes can be rescued by *inc*-spanning genomic duplications. (A) The schematic shows the knock-in strategy for creating *inc^mw^*, genomic location of *inc*, 3 *inc-*spanning duplications (Dp), and a non-*inc-*spanning duplication, exon/intron structure of *inc*, insertion sites of *inc^f00285^* and *inc^mw^*, and regions of homology of 2 *UAS-inc-RNAi* lines of interest. (B) Sleep duration, (C) average sleep bout length (ABL), (D) number of sleep bouts in rescue of *inc^f00285^* with genomic duplications. Error bars are SEM. n>30 male flies. * p<0.005 with Student's t test.
(PDF)

Figure S3 Short, poorly-consolidated sleep in *inc* females. (A) Sleep duration and (B) average sleep bout length (ABL) for *inc^f00285^* homozygotes (n = 50 females), *inc^mw^* homozygotes (n = 42 females), *inc^f00285^/inc^mw^* transhets (n = 40 females), *inc^f00285^* over an *inc*-spanning deficiency (*f00285/Df* n = 30 females), *inc^f00285^*/+ (n = 23 females), *inc^mw^*/+ (n = 38 females), deficiency heterozygotes (+/Df n = 44 females), and. *iso31* control (n = 26 females). Error bars are SEM. * p<0.03 with Student's t test.
(PDF)

Figure S4 *inc^f00285^* flies have a reduced homeostatic response after 24 h mechanical sleep deprivation. The graph shows that reduced homeostatic response to sleep loss in *inc^f00285^* is not due to a lower magnitude sleep loss as compared to wild type. Sleep loss after 24 h sleep deprivation in *inc^f00285^* is comparable to 12 h sleep deprivation in wild type. The red is cumulative Δsleep (min) during mechanical sleep deprivation, light blue is 6 h rebound after deprivation, dark blue is 9 h. n>20 females for each genotype. * p<0.05. Error bars are SEM.
(PDF)

Figure S5 *inc^f00285^ Gal4* rescue screen. (A) Sleep duration and (B) average sleep bout length (ABL) for *Gal4* rescue of *inc^f00285^* (red) and *Gal4* heterozygotes,(i.e., no UAS, wild-type controls; grey) in males. Arrows highlight *Gal4* lines that rescue the *inc^f00285^* sleep phenotype (p<0.01 with Student's t test). n>20 males for all genotypes except *inc^f00285^*×Hdc-G4 (n = 8, semi-lethal). Error bars are SEM.
(PDF)

Figure S6 Expression pattern of *Gal4s* that rescue *inc^f00285^*. (A–B) *30Y-Gal4*×*UAS-mGFP* in (A) an adult male brain and (B) an L3 male CNS. (C–D) *G0451-Gal4*×*UAS-mGFP* in (C) an adult male brain and (D) an L3 male CNS. (E–F) *c309-Gal4*×*UAS-mGFP* in (E) an adult male brain and (F) an L3 male CNS. (G–H) *Cha-Gal4*×*UAS-mGFP* in (G) an adult male brain and (H) an L3 male CNS. Scale bars are 100 µm.
(PDF)

Figure S7 RNAi knockdown of *Cul3* with a second *Cul3-RNAi* line in post-mitotic neurons alters sleep architecture. Sleep duration (A), latency to sleep after lights-off (B), and average sleep bout length (ABL) (C) with a second *Cul3-RNAi* knockdown line and *UAS-Cul3* rescue in post-mitotic neurons in males (n>26 male flies for all conditions). (D) Pan-neuronal knockdown with the second *Cul3-RNAi* line results in reduced *Cul3* transcript levels as compared with heterozygous controls. Error bars are SEM. ** p<<0.001, * p<0.05.
(PDF)

Figure S8 Gross morphology of sleep-relevant brain regions is normal in *inc* mutants. Micrographs compare (A) *pars intercerebralis* morphology as labeled with *Takr86c-LexA×LexAop-mGFP*, (B) PDF cell morphology with α-PDF immunostaining, and (C) dopaminergic cell morphology as labeled with α-TH immunostaining between *iso31*, *inc^f00285^*, and *inc^mw^* control adult male brains. In each case the expression patterns are indistinguishable. Scale bars are 100 µm.
(PDF)

Figure S9 Stochastic mushroom body branching defects in *inc* mutants. Micrographs show mushroom body (MB) morphology as visualized with α-FASII (green) and 247dsRed (red) expression patterns. (A) 15 of 15 *iso31* brains exhibit normal MB morphology. (B) 40 of 50 *inc^f00285^* brains have normal MB morphology (B1); however, 10 lack either a single α or β lobe (B2). (C) 25 of 38 *inc^mw^* brains have normal MB morphology (C1); however, 13 lack either a single α or β lobe (C2). (D) 15 of 15 *Sh^mns^* brains have normal MB morphology. (E) 10 of 10 *DAT^fmn^* brains have normal MB morphology. (F–G) The graphs show the percentile distribution of sleep duration (F) and ABL (G) phenotypes in *inc^f00285^* (n = 44 males, red), *inc^mw^* (n = 72 males, dark blue), and *iso31* (n = 54 males, grey). The purple dotted line marks the lowest *iso31* fly, the green dotted line marks the 20th percentile fly. Scale bar is 100 µm. Error bars are SEM.
(PDF)

Figure S10 *inc* functions in a dopaminergic arousal pathway. (A–B) Graphs show sleep duration in *inc^f00285^*, *inc^mw^*, and *iso31* after consumption of food laced with the *Rdl* antagonist 0.25 mg/mL CBZ (A; purple) or 5 mg/mL L-DOPA (B; yellow; n>31 males for all conditions). Error bars are SEM. ** p<0.001 with Student's t test.
(PDF)

Figure S11 Head dopamine levels are altered by consumption of 3IY and L-DOPA. (A) Graph shows head dopamine levels in *iso31* males after 3 d on regular food (grey) or food laced with 2 mg/mL 3IY (green). (B) The graph shows head dopamine levels in *iso31* male flies after 3 d on regular food (grey), food laced with 2 mg/mL, or 5 mg/mL L-DOPA. (C) Western blot shows head TH levels are indistinguishable between *inc^f00285^* and *iso31* males. Error bars are SEM. * p<0.001 with Student's t test.
(PDF)
