# Supplementary figures and images for: Cul3 and the BTB Adaptor Insomniac Are Key Regulators of Sleep Homeostasis and a Dopamine Arousal Pathway in Drosophila
Source: PLoS Genet. 2012 Oct 4;8(10):e1003003. doi: 10.1371/journal.pgen.1003003 (PMC3464197; doi:10.1371/journal.pgen.1003003)

**Figure S1: Genetic background modulates sleep phenotypes.**

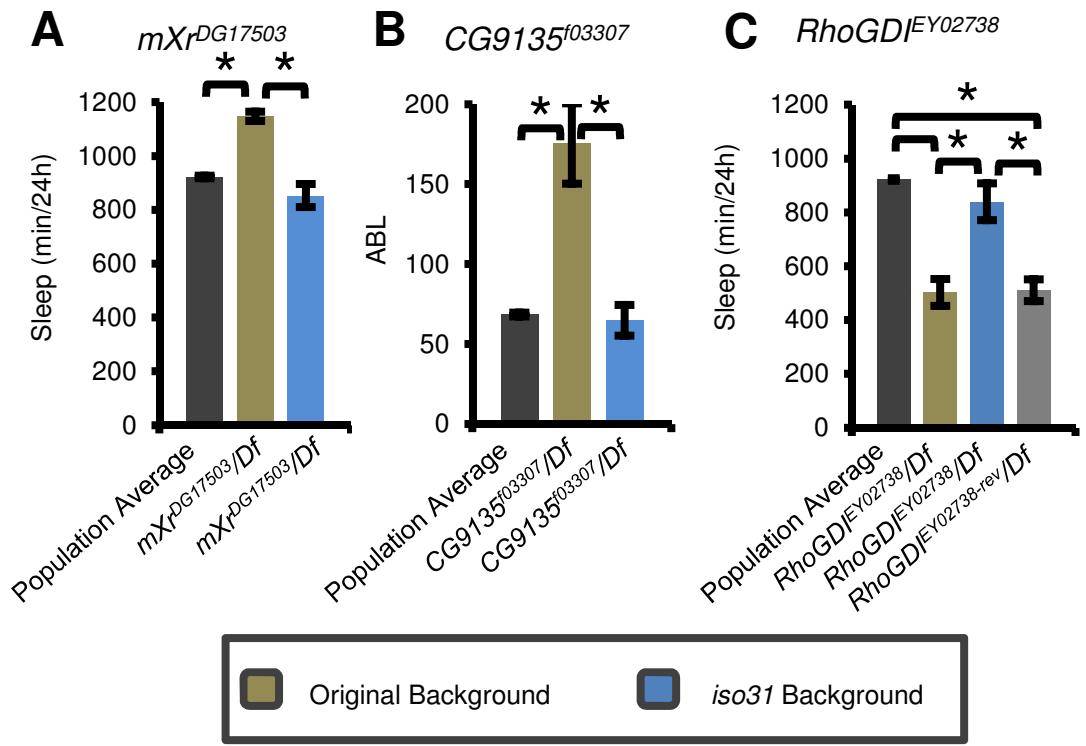

Supplement: Figure S1 — Genetic background modulates sleep phenotypes. (A) Sleep duration in mXrDG17503/Df(2R)ED1742 in the original genetic background (tan, n = 20 males) as compared to the iso31 background (blue, n = 10 males) and the screen population average (grey, >4000 males). (B) Average sleep bout length (ABL) in CG9135f03307/Df(2L)ED353 in the original genetic background (tan, n = 20 males) as compared to the iso31 background (blue, n = 10 males) and the screen population average (grey, >4000 males). (C) Sleep duration in RhoGDIEY02738/Df(3L)ED4858 in the original genetic background (tan, n = 20 males) as compared to the iso31 background (blue, n = 10 males), the screen population average (grey, >4000 males) and a precise excision revertant (RhoGDIEY02738-rev, light grey, n = 10 males). Error bars are SEM. * p<0.001. (PDF) [file pgen.1003003.s001.pdf]

Figure S2: *inc*<sup>f00285</sup> sleep phenotypes can be rescued by *inc*-spanning genomic duplications.

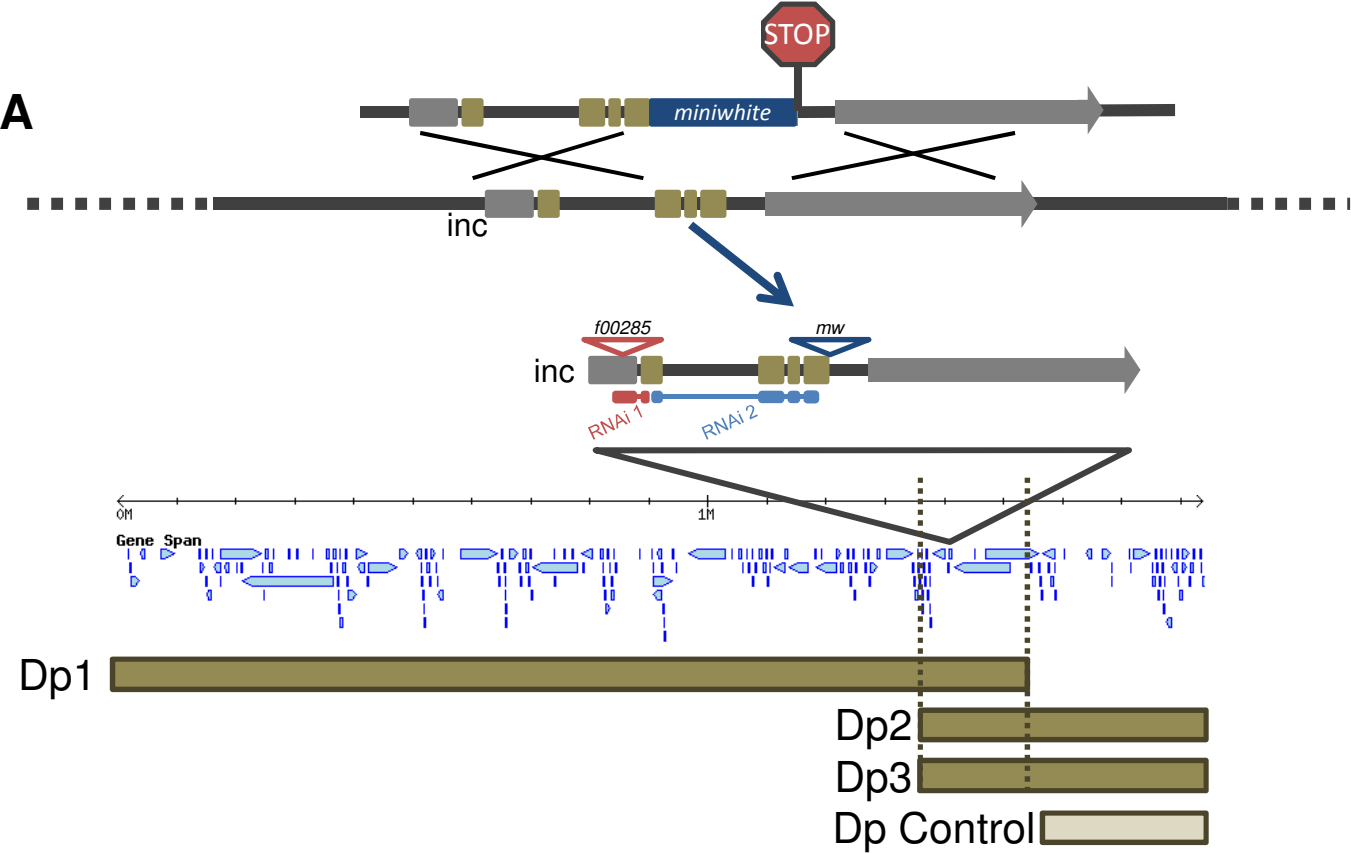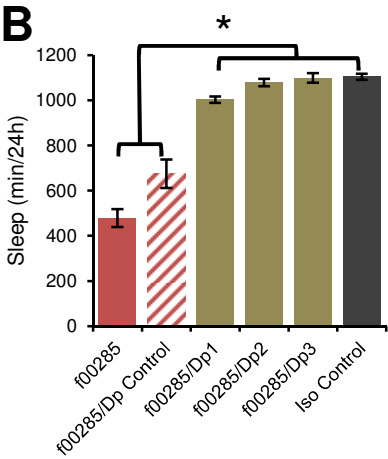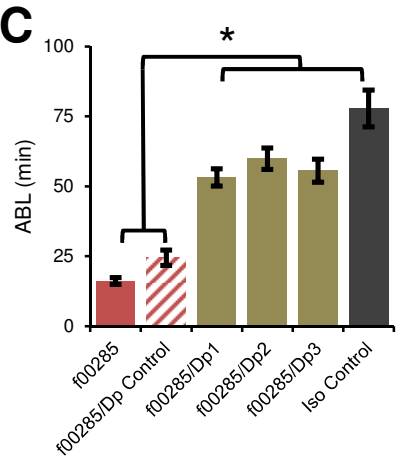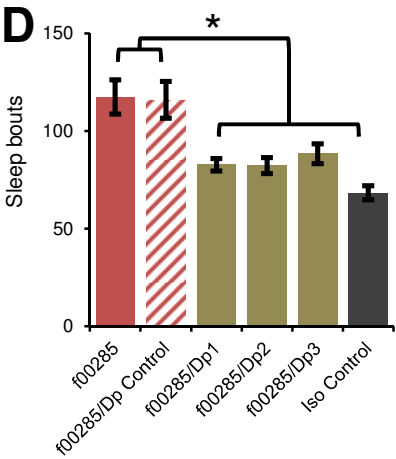

Supplement: Figure S2 — incf00285 sleep phenotypes can be rescued by inc-spanning genomic duplications. (A) The schematic shows the knock-in strategy for creating incmw, genomic location of inc, 3 inc-spanning duplications (Dp), and a non-inc-spanning duplication, exon/intron structure of inc, insertion sites of incf00285 and incmw, and regions of homology of 2 UAS-inc-RNAi lines of interest. (B) Sleep duration, (C) average sleep bout length (ABL), (D) number of sleep bouts in rescue of incf00285 with genomic duplications. Error bars are SEM. n>30 male flies. * p<0.005 with Student's t test. (PDF) [file pgen.1003003.s002.pdf]

Figure S3: Short, poorly-consolidated sleep in *inc* females.

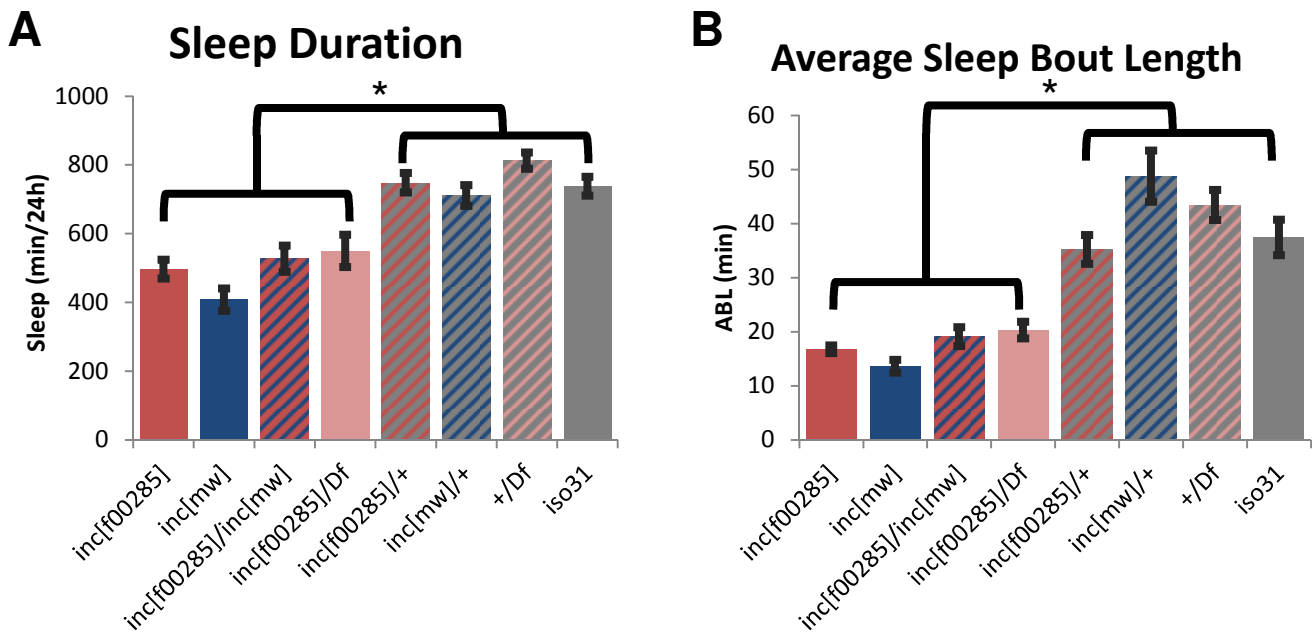

Supplement: Figure S3 — Short, poorly-consolidated sleep in inc females. (A) Sleep duration and (B) average sleep bout length (ABL) for incf00285 homozygotes (n = 50 females), incmw homozygotes (n = 42 females), incf00285/incmw transhets (n = 40 females), incf00285 over an inc-spanning deficiency (f00285/Df n = 30 females), incf00285/+ (n = 23 females), incmw/+ (n = 38 females), deficiency heterozygotes (+/Df n = 44 females), and. iso31 control (n = 26 females). Error bars are SEM. * p<0.03 with Student's t test. (PDF) [file pgen.1003003.s003.pdf]

Figure S5: *inc<sup>f00285</sup>* *Gal4* rescue screen.

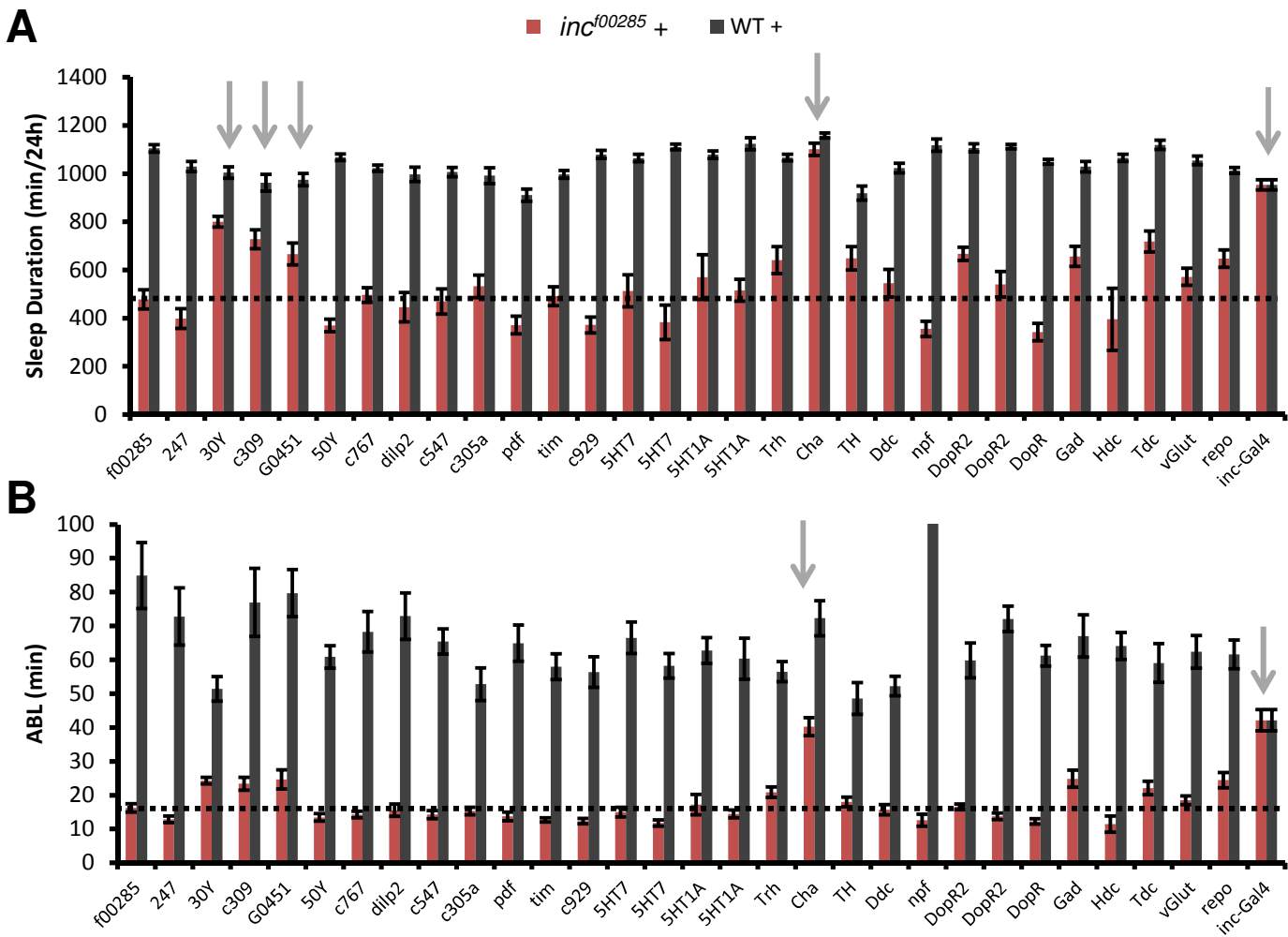

Supplement: Figure S5 — incf00285 Gal4 rescue screen. (A) Sleep duration and (B) average sleep bout length (ABL) for Gal4 rescue of incf00285 (red) and Gal4 heterozygotes,(i.e., no UAS, wild-type controls; grey) in males. Arrows highlight Gal4 lines that rescue the incf00285 sleep phenotype (p<0.01 with Student's t test). n>20 males for all genotypes except incf00285×Hdc-G4 (n = 8, semi-lethal). Error bars are SEM. (PDF) [file pgen.1003003.s005.pdf]

Figure S6: Expression pattern of *Gal4s* that rescue *inc*<sup>f00285</sup>.

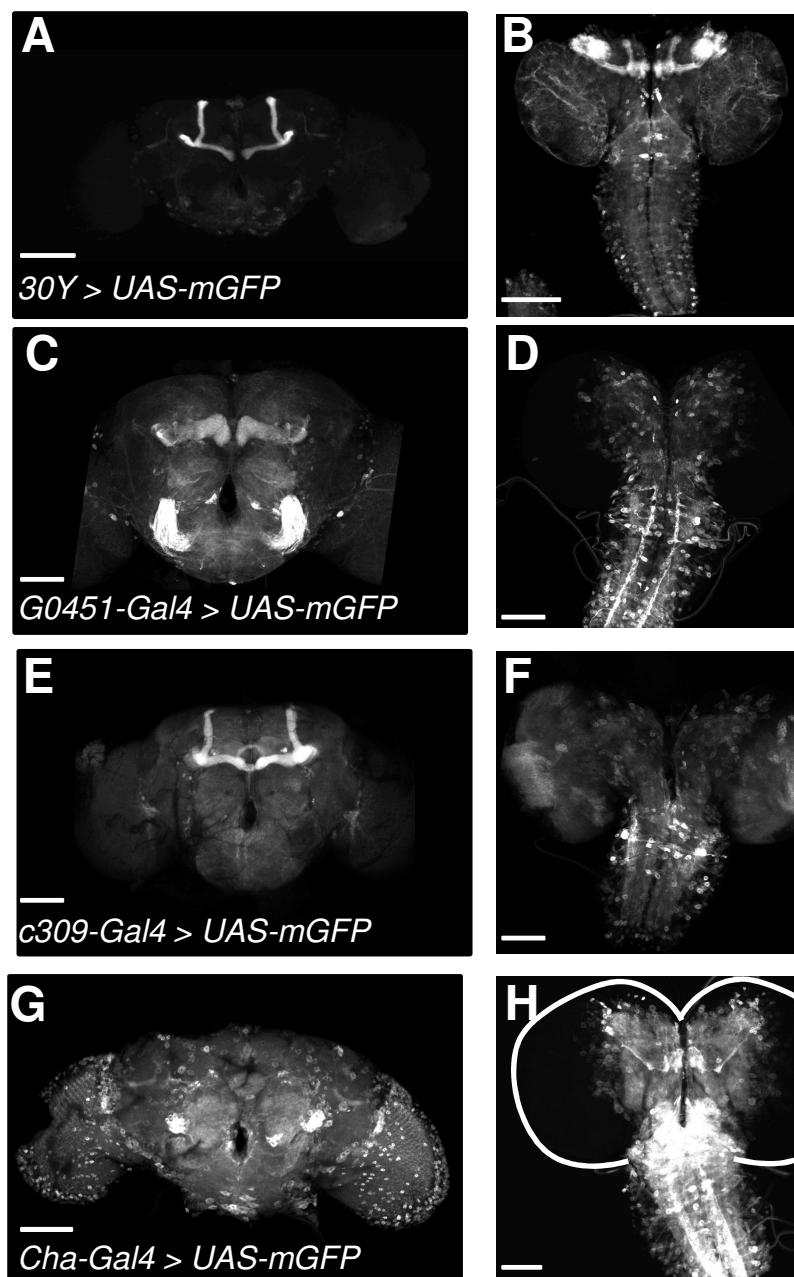

Supplement: Figure S6 — Expression pattern of Gal4s that rescue incf00285. (A–B) 30Y-Gal4×UAS-mGFP in (A) an adult male brain and (B) an L3 male CNS. (C–D) G0451-Gal4×UAS-mGFP in (C) an adult male brain and (D) an L3 male CNS. (E–F) c309-Gal4×UAS-mGFP in (E) an adult male brain and (F) an L3 male CNS. (G–H) Cha-Gal4×UAS-mGFP in (G) an adult male brain and (H) an L3 male CNS. Scale bars are 100 µm. (PDF) [file pgen.1003003.s006.pdf]

**Figure S8: Gross morphology of sleep-relevant brain regions is normal in *inc* mutants.**

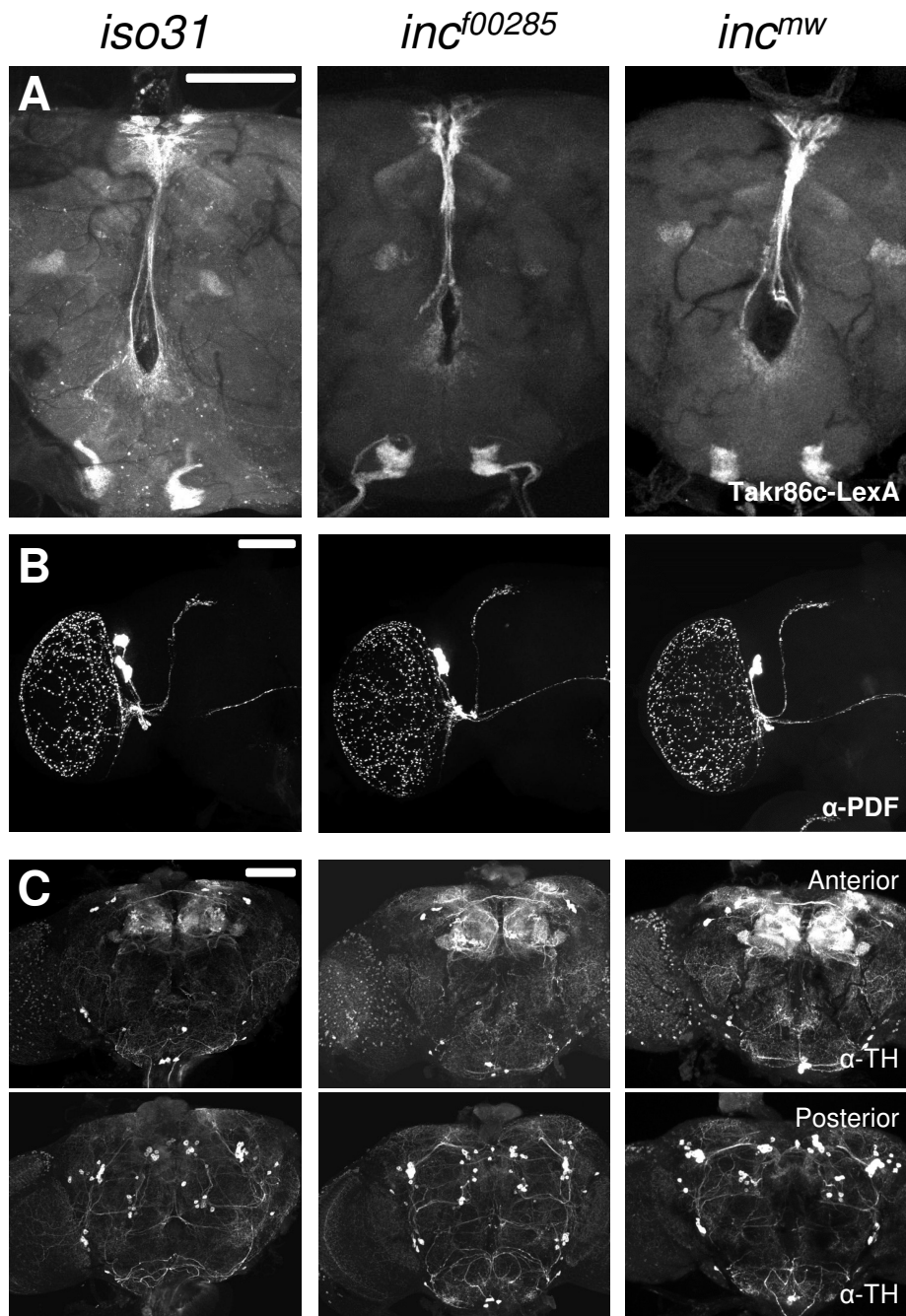

Supplement: Figure S8 — Gross morphology of sleep-relevant brain regions is normal in inc mutants. Micrographs compare (A) pars intercerebralis morphology as labeled with Takr86c-LexA×LexAop-mGFP, (B) PDF cell morphology with α-PDF immunostaining, and (C) dopaminergic cell morphology as labeled with α-TH immunostaining between iso31, incf00285, and incmw control adult male brains. In each case the expression patterns are indistinguishable. Scale bars are 100 µm. (PDF) [file pgen.1003003.s008.pdf]

Figure S9: Stochastic mushroom body branching defects in *inc* mutants.

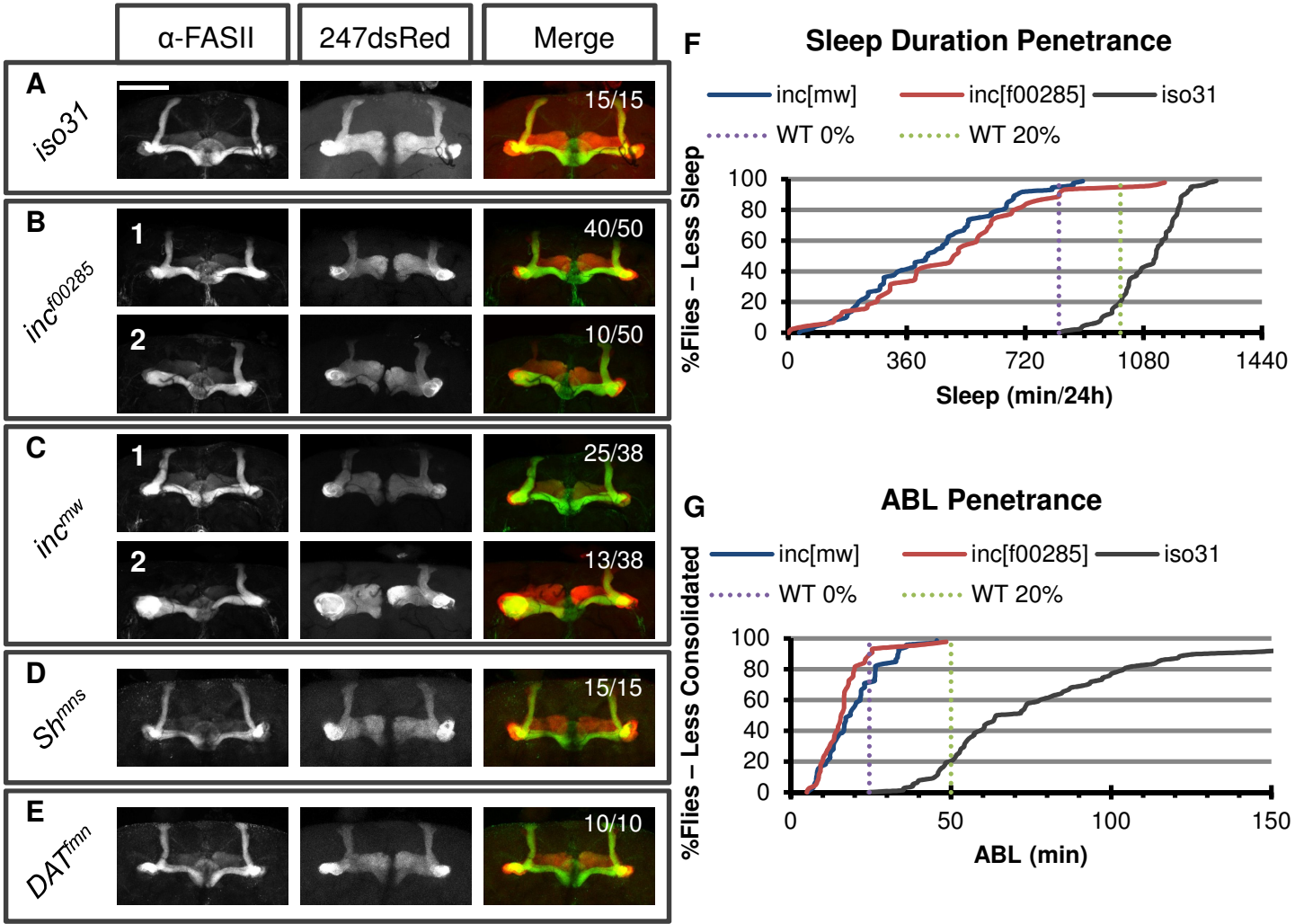

Supplement: Figure S9 — Stochastic mushroom body branching defects in inc mutants. Micrographs show mushroom body (MB) morphology as visualized with α-FASII (green) and 247dsRed (red) expression patterns. (A) 15 of 15 iso31 brains exhibit normal MB morphology. (B) 40 of 50 incf00285 brains have normal MB morphology (B1); however, 10 lack either a single α or β lobe (B2). (C) 25 of 38 incmw brains have normal MB morphology (C1); however, 13 lack either a single α or β lobe (C2). (D) 15 of 15 Shmns brains have normal MB morphology. (E) 10 of 10 DATfmn brains have normal MB morphology. (F–G) The graphs show the percentile distribution of sleep duration (F) and ABL (G) phenotypes in incf00285 (n = 44 males, red), incmw (n = 72 males, dark blue), and iso31 (n = 54 males, grey). The purple dotted line marks the lowest iso31 fly, the green dotted line marks the 20th percentile fly. Scale bar is 100 µm. Error bars are SEM. (PDF) [file pgen.1003003.s009.pdf]

Figure S10: *inc* functions in a dopaminergic arousal pathway.

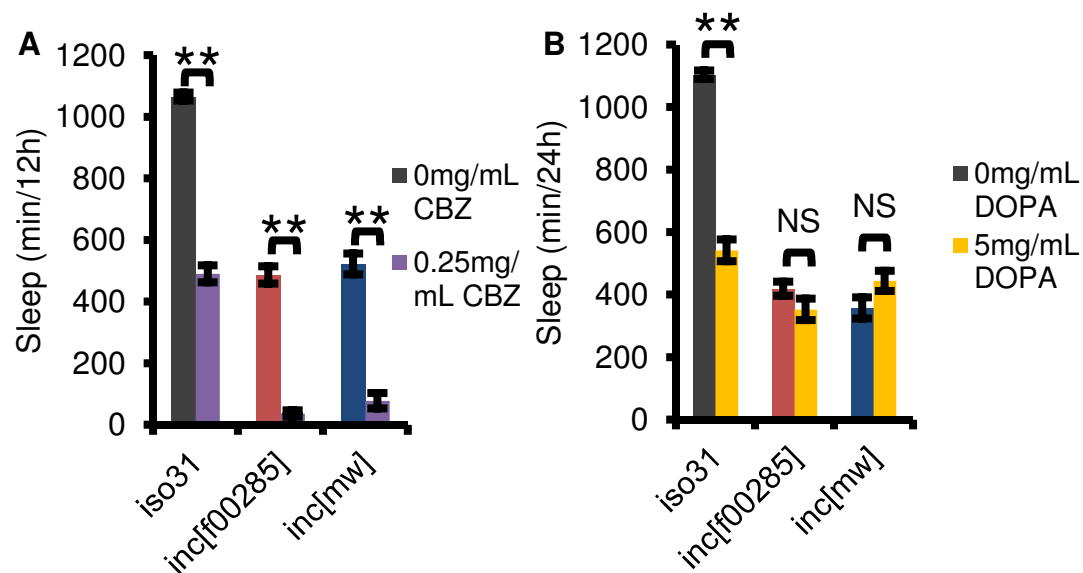

Supplement: Figure S10 — inc functions in a dopaminergic arousal pathway. (A–B) Graphs show sleep duration in incf00285, incmw, and iso31 after consumption of food laced with the Rdl antagonist 0.25 mg/mL CBZ (A; purple) or 5 mg/mL L-DOPA (B; yellow; n>31 males for all conditions). Error bars are SEM. ** p<0.001 with Student's t test. (PDF) [file pgen.1003003.s010.pdf]

Figure S11: Head dopamine levels are altered by consumption of 3IY and L-DOPA.

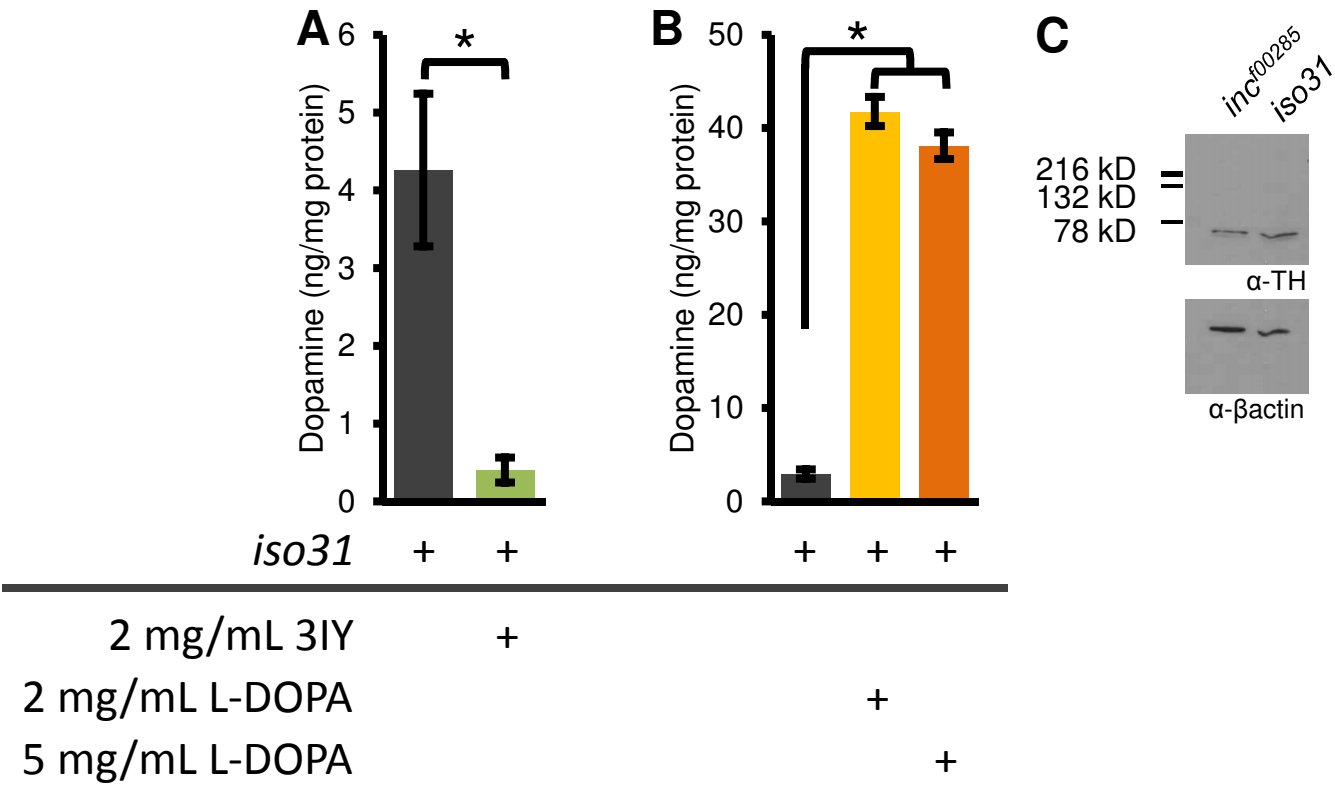

Supplement: Figure S11 — Head dopamine levels are altered by consumption of 3IY and L-DOPA. (A) Graph shows head dopamine levels in iso31 males after 3 d on regular food (grey) or food laced with 2 mg/mL 3IY (green). (B) The graph shows head dopamine levels in iso31 male flies after 3 d on regular food (grey), food laced with 2 mg/mL, or 5 mg/mL L-DOPA. (C) Western blot shows head TH levels are indistinguishable between incf00285 and iso31 males. Error bars are SEM. * p<0.001 with Student's t test. (PDF) [file pgen.1003003.s011.pdf]
