# Supplementary material for: Cul3 and the BTB Adaptor Insomniac Are Key Regulators of Sleep Homeostasis and a Dopamine Arousal Pathway in Drosophila
Source: PLoS Genet. 2012 Oct 4;8(10):e1003003. doi: 10.1371/journal.pgen.1003003 (PMC3464197; doi:10.1371/journal.pgen.1003003)

Figure S4: *inc*^{f00285} flies have a reduced homeostatic response after 24h mechanical sleep deprivation.

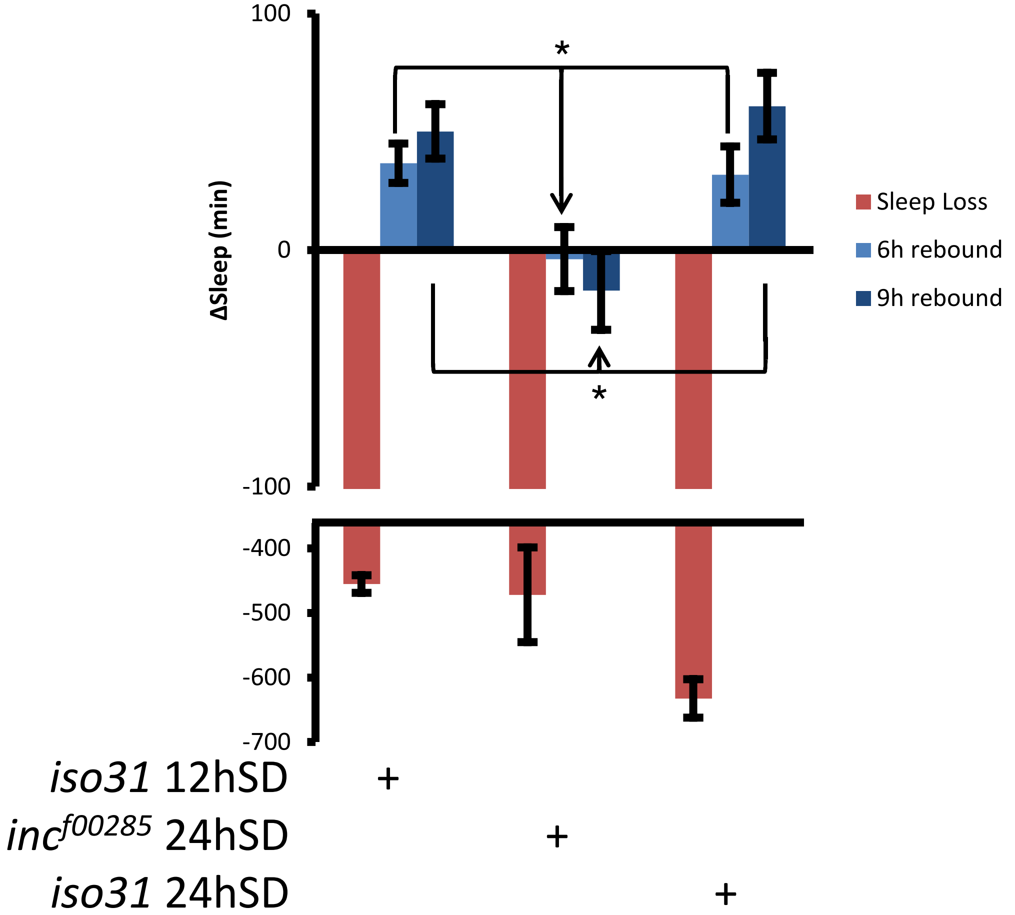

Supplement: Figure S4 — incf00285 flies have a reduced homeostatic response after 24 h mechanical sleep deprivation. The graph shows that reduced homeostatic response to sleep loss in incf00285 is not due to a lower magnitude sleep loss as compared to wild type. Sleep loss after 24 h sleep deprivation in incf00285 is comparable to 12 h sleep deprivation in wild type. The red is cumulative Δsleep (min) during mechanical sleep deprivation, light blue is 6 h rebound after deprivation, dark blue is 9 h. n>20 females for each genotype. * p<0.05. Error bars are SEM. (PDF) [file pgen.1003003.s004.pdf]
