# Supplementary material for: Cul3 and the BTB Adaptor Insomniac Are Key Regulators of Sleep Homeostasis and a Dopamine Arousal Pathway in Drosophila
Source: PLoS Genet. 2012 Oct 4;8(10):e1003003. doi: 10.1371/journal.pgen.1003003 (PMC3464197; doi:10.1371/journal.pgen.1003003)

Figure S7: RNAi knockdown of *Cul3* with a second *Cul3-RNAi* line in post-mitotic neurons alters sleep architecture.

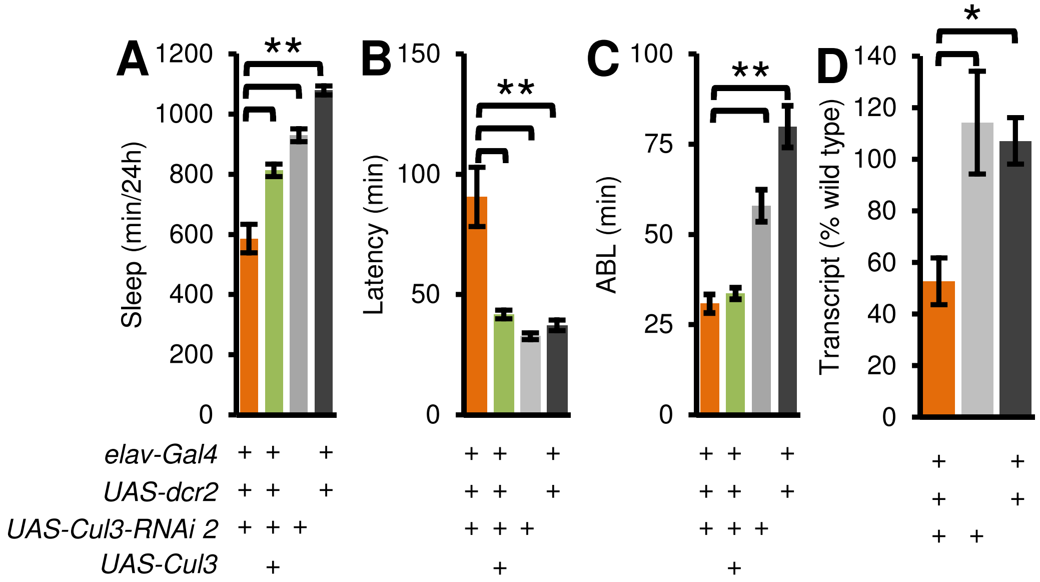

Supplement: Figure S7 — RNAi knockdown of Cul3 with a second Cul3-RNAi line in post-mitotic neurons alters sleep architecture. Sleep duration (A), latency to sleep after lights-off (B), and average sleep bout length (ABL) (C) with a second Cul3-RNAi knockdown line and UAS-Cul3 rescue in post-mitotic neurons in males (n>26 male flies for all conditions). (D) Pan-neuronal knockdown with the second Cul3-RNAi line results in reduced Cul3 transcript levels as compared with heterozygous controls. Error bars are SEM. ** p<<0.001, * p<0.05. (PDF) [file pgen.1003003.s007.pdf]
